# Anti-platelet aggregation activities of different grades of *Angelica sinensis* and their therapeutic mechanisms in rats with blood deficiency: insights from metabolomics and lipidomics analyses

**DOI:** 10.3389/fphar.2023.1230861

**Published:** 2024-01-03

**Authors:** Xue Shen, Yangyang Wu, Ping Chen, Yuwei Bai, Yanan Liu, Yihan Jiang, Yawen Zhang, Zhigang Yang

**Affiliations:** ^1^ School of Pharmacy, Lanzhou University, Lanzhou, China; ^2^ Collaborative Innovation Center for Northwestern Chinese Medicine, Lanzhou University, Lanzhou, China; ^3^ State Key Laboratory of Applied Organic Chemistry, Lanzhou University, Lanzhou, China

**Keywords:** *Angelica sinensis*, different commodity grades, anti-platelet aggregation activity, blood deficiency rat, Metabolomics, lipidomics, Q-marker

## Abstract

In traditional Chinese medicine, the radix of *Angelica sinensis* (Oliv.) Diels (RAS) is mainly used to replenish and invigorate the blood circulation. This study investigated anti-platelet aggregation activities were used by New Zealand rabbits, and high-performance liquid chromatography data were obtained to determine the spectrum–effect relationship for different commercial grades of RAS. Plasma and urine metabolites were examined using ultra-performance liquid chromatography coupled with quadrupole time-of-flight tandem mass spectrometry-based metabolomics to elucidate the mechanisms underlying the role of these metabolites in a rat model of blood deficiency (BD). Plasma and spleen metabolites were additionally examined using ultra-performance liquid chromatography plus Q-Exactive tandem mass spectrometry-based lipidomics to clarify the mechanisms of RAS in treating BD. The third grade of RAS exhibited the best activity in replenishing and invigorating blood *in vitro* and *in vivo*. Ferulic acid, ligustilide, senkyunolide I, uridine, and guanine are quality markers of anti-platelet aggregation activity. Based on the metabolomics results, 19 potential biomarkers were screened in plasma, and 12 potential metabolites were detected in urine. In lipidomics analyses, 73 potential biomarkers were screened in plasma, and 112 potential biomarkers were screened in the spleen. RAS may restore lipid metabolism by regulating disorders of glycerophospholipid and sphingolipid metabolism, the tricarboxylic acid cycle, amino acid metabolism (thereby improving energy metabolism), and arachidonic acid metabolism (thereby promoting blood circulation). These results provide a deeper understanding of the effects of different grades of RAS and a scientific reference for the establishment of grading standards and for the clinical use of RAS.

## 1 Introduction

The raw materials employed in traditional Chinese medicine (TCM) exhibit a dual nature, serving as medicinal components and commercial commodities. The commercial-grade specification of TCM components plays a vital role in determining a product’s medicinal value within the market (Wei et al., 2022). To ensure the quality and premium pricing of TCM materials, it has become imperative to enhance the criteria and standards for these commodities (Qian et al., 2019). The critical commodity classification method, referred to as “distinguishing appearance and quality,” assesses various attributes such as shape, size, color, surface characteristics, texture, section features, and scent to gauge quality (He et al., 2022). However, it is often the case that there is no clear scientific correlation between commodity grade specifications and the bioactivities of TCM raw materials. For instance, low-grade *Panax notoginseng* exhibits superior hemostatic activity compared to its high-grade counterpart (Liu et al., 2016). Consequently, the accurate utilization of high-quality Chinese herbal medicines presents a significant challenge that must be addressed to ensure optimal clinical efficacy.

The radix of *Angelica sinensis* (Oliv.) Diels (RAS) replenishes and invigorates blood circulation, regulates menstruation, relieves pain, and moistens the intestines, as claimed by the Pharmacopoeia of the People’s Republic of China in 2020. RAS is categorized into different grades based on weight, as specified by T/CACM in 2018. RAS of the first to fourth grades was used in this study. It has been discovered that there is a positive correlation between blood tonic activity and RAS weight, a negative correlation between polysaccharide and RAS weight, and no association between ligustilide and RAS weight (Yang, 2017).

The clinical signs of hemolytic anemia in TCM include pale skin, a white tongue, palpitations, dizziness, and frequent bleeding (Hwang et al., 2022). A combination of acetylphenylhydrazine (APH) and cyclophosphamide (CTX) has been used to develop models of blood deficiency (BD) (Song et al., 2014). Previous studies have demonstrated that RAS can enhance peripheral blood indices in rat models of BD (Hua et al., 2017). RAS extracts have also been found to significantly inhibit platelet aggregation (Chen et al., 2019). However, there is no evidence for variance in effectiveness or differences in the therapeutic mechanisms among the different grades of RAS in replenishing and invigorating blood circulation.

Metabolomics examines the variations in small-molecule metabolites produced under various intervention conditions in order to detect the differences among creatures’ metabolisms after treating with TCM (Cui et al., 2022). Herbs are multifaceted components with diverse targeting properties. Research has demonstrated the principles underlying TCM treatments using metabolomics in numerous studies (Chang et al., 2016). Additionally, lipidomics plays a crucial role in identifying key lipid biomarkers in metabolic regulation and in elucidating the roles of lipids in various physiological processes (Yang and Han, 2016).

In this study, we investigated the effects of various grades of RAS in terms of replenishing and invigorating blood circulation, both *in vitro* and *in vivo*. We employed untargeted metabolomics and lipidomic profiling of plasma, urine, and spleen samples to unveil potential therapeutic mechanisms.

## 2 Material and methods

### 2.1 Reagents and materials

RAS was collected from Min Country of Gansu Province, China, in November 2020. All of the plant materials were authenticated as *A. sinensis* (Oliv.) Diels by Professor Zhigang Yang. Polymerase chain reaction (PCR) tests were performed to identify the RAS (Liu et al., 2018). The PCR results are shown in Supplementary Figure S1. Voucher specimens (No. DG202009001) were stored at the School of Pharmacy, Lanzhou University. The weights per RAS for specimens falling into the first to fourth grades were more than 60.0 g, 25.0–60.0 g, 15.0–25.0 g, and 10.0–15.0 g, respectively.

Ferulic acid, ligustilide, senkyunolide I, chlorogenic acid, levistolide A, uridine, adenosine, guanine, and *L*-tryptophan were purchased from Chengdu Pufeide Biotech Co., Ltd. (Chengdu, China) as reference compounds. Acetonitrile and Methanol were purchased from CNW Technologies GmbH. Formic acid was purchased from Merck (Darmstadt, Germany). Double-distilled water (ddH_2_O) was obtained using a laboratory water purifier. APH, CTX, and ATPase were purchased from Beijing Solarbio Science and Technology Co., Ltd. (Beijing, China). Adenosine diphosphate (ADP) and arachidonic acid (AA) were purchased from Shandong Tailixin Medical Technology Co., Ltd. (Shandong, China). Finally, aspirin was provided by the CSPC Pharmaceutical Group Co., Ltd.

### 2.2 Metabolite extraction from RAS and drug administration

RAS powders (100.0 g) of different grades were weighed and extracted with 1,000 mL of deionized water by refluxing in a hot water bath for 1 h. Subsequently, the sediment was filtered and soaked for 1 h in an 8-fold deionized water for the second extraction. The obtained filtrates were mixed and concentrated. The concentration of the water extracts of RAS used in BD model rats was 7.5 mg/mL (7.5 mg of RAS per milliliter of deionized water). Reference compounds were weighed and dissolved in ddH_2_O to a final concentration of 5 mg/mL for high-performance liquid chromatography (HPLC).

### 2.3 Animals and preparation of the BD model

Healthy New Zealand rabbits (20 weeks, 2.5–3.0 kg) and male Wistar rats (8 weeks, 180 ± 20 g) were obtained from the Animal Center of Lanzhou University. All animal procedures were performed in accordance with the Guidelines for the Care and Use of Laboratory Animals of Lanzhou University and were approved by the Animal Ethics Committee of Lanzhou University: SCXK (gan) 2018–0002.

The healthy male Wistar rats were initially allowed to freely consume water and food under constant temperature (24°C ± 2°C), humidity (70% relative humidity), and light–dark cycles (12 h light/dark) in the Specific Pathogen Free Laboratory. The rats were fed standard commercial chow and purified water was available *ad libitum*. After 1 week of acclimation, 81 Wistar rats were randomly divided into nine groups with nine rats in each: 1) a control group (C); 2) the model group (M); 3) the aspirin group (P, positive control drug, 7.5 mg/mL); 4) the first-grade RAS group (F, 7.5 mg/mL); 5) the second-grade RAS group (S, 7.5 mg/mL); (6) the third-grade RAS group (T, 7.5 mg/mL); 7) the fourth-grade RAS group (Fo, 7.5 mg/mL); 8) the high-dose third-grade RAS group (H, 15 mg/mL); and 9) the low-dose third-grade RAS group (L, 3.5 mg/mL). As the clinical dosage of RAS is 6–12 g per day (Committee, 2020), the clinically equivalent dose was calculated according to the animal experimental dose. The animal drug dose calculated based on the body mass of the RAS decoction was 0.01 mL/g, and oral gavage was administered once daily continuously for 7 days from day 1. The rats in the control and model groups were administered an equal volume of normal saline. The M, P, F, S, T, and Fo groups were hypodermically injected with 4 mg/mL APH saline solution on days 1–3 at a dose of 20 mg/kg. After a 2 h hypodermic injection on day 3, the rats were intraperitoneally injected with 4 mg/mL CTX saline solution on days 3–6 at 20 mg/kg. All the rats were allowed free access to feed and water during the experiment, and the entire experiment lasted for 7 days. Their body weight was measured and recorded daily during the experiment.

### 2.4 High-performance liquid chromatography analysis of water extracts of RAS

All water extracts of RAS were analyzed using an Agilent 1260 HPLC system (Agilent Technologies, Santa Clara, CA, United States). Separation was carried out on an HPLCONE-5C18D column (250 mm × 4.6 mm, 5 µm), which was maintained at 30°C and eluted at a flow rate of 1 mL/min. The mobile phase consisted of water containing 0.1% formic acid (A) and acetonitrile (B) for gradient elution. A linear gradient of 0–5 min, 3%–10% (B); 5–15 min, 15% (B); 15–25 min, 25% (B); 25–35 min, 35% (B); 35–40 min, 70% (B); 40–55 min, 95% (B); 55–60 min, 100% (B) was used. The injection volume and detection wavelength were 10 μL and 280 nm, respectively.

### 2.5 Anti-platelet aggregation assay

The anti-platelet aggregation assay was carried out according to methods reported in our previous research (Zhang et al., 2022a).

### 2.6 Peripheral hemogram assay and histopathology analysis

Twelve hours after the last administration, blood was taken from the abdominal aorta, and the plasma was separated into several portions (200 μL each) and stored at −80°C for further analysis. Levels of red blood cells (RBC, 10^12^/L), white blood cells (WBC, 10^9^/L), hemoglobin (HGB, g/L), hematocrit (HCT, %), platelets (PLT, 10^9^/L), lymphocytes (LY, %), monocytes (MO, %) and neutrophils (NE, %) were also examined using an auto-hemocytome with a Hemavet 950FS automatic blood analyzer (Ellis, Beijing, China). After the animals were euthanized, their organs were separated, fixed in formalin, and embedded in paraffin. Samples were cut into thin slices and stained with hematoxylin–eosin (HE). Organ histological changes were observed using a BX53 light microscope (Olympus, Tokyo, Japan). Spleen samples were collected and weighed. Spleen indices were calculated using the organ index formula (organ index (g/g) = organ weight/body weight), and spleen indices were calculated. All organ samples were kept at −80°C until analysis.

### 2.7 Determination of ATPase content in the erythrocyte membrane

The ATPase content in the rat erythrocyte membranes was measured according to the instructions of the ATPase kit.

### 2.8 Untargeted metabolomics analysis

For rat plasma samples, approximately 200 μL was mixed with 800 μL of acetonitrile in a 2-mL conical tube. After vigorous vortexing for 2 min and subsequent centrifugation (15 min, 15,000 rpm, 4°C), the supernatant was filtered through a 0.22-μm membrane filter. The filtered liquid was then evaporated using a nitrogen blower. Subsequently, 25 μL of 40% acetonitrile was introduced to redissolve the samples. Following another round of vortexing (2 min) and centrifugation (15 min, 15,000 rpm, 4°C), the supernatant was collected for further analysis.

For urine samples, approximately 1.2 mL was mixed with 3.6 μL of acetonitrile in a 10-mL conical tube. After thorough vortexing for 2 min and subsequent centrifugation (15 min, 15,000 rpm, 4°C), the supernatant was evaporated using a nitrogen blower. Next, 400 μL of acetonitrile (40%) was added to redissolve the samples. After another round of vortexing (2 min) and centrifugation (15 min, 15,000 rpm, 4°C), the supernatant was collected and prepared for filtering and analysis.

To ensure the stability of the analytical methods and instruments, quality control (QC) samples were prepared using 5 μL from each plasma sample. These QC samples were analyzed alongside every set of six samples throughout the entire analysis process.

Sample analysis was carried out using an Agilent 6560 QTOF mass spectrometer (Agilent Technologies, Santa Clara, CA, United States). Chromatographic separation was achieved with an ACQUITY BEH C18 column (2.1 mm × 50 mm, 1.7 µm, Waters, United States) maintained at 40°C. The mobile phase consisted of water containing 0.1% formic acid (A) and acetonitrile containing 0.1% formic acid (B), following gradient elution at a 0.3 mL/min flow rate. A linear gradient sequence was applied as follows: 0–1 min, 5% (B); 1–6 min, 5%–20% (B); 6–9 min, 20%–50% (B); 9–15 min, 50%–95% (B); 15–20 min, 95% (B); 20–23 min, 95%–100% (B); 23–25 min, 100%–5% (B); 25–30 min, 5% (B). The injection volume and detection wavelength were 4 nm and 280 nm, respectively.

The ion-source parameters were optimized and set as follows: capillary voltage, 3,500 V; drying gas temperature, 225°C; drying gas flow rate, 10 L/min; nebulizer pressure, 25 psig; sheath gas temperature, 400°C; sheath gas flow rate, 12 L/min. Data were acquired at a rate of 1 spectrum/s with a scanning range of 20–1,500 m*/z*.

### 2.9 Untargeted lipidomic analysis

For plasma samples, approximately 40 μL was mixed with 300 μL of acetonitrile in a 2-mL conical tube and vortex-shaken for 1 min. Subsequently, 1,000 μL of methyl tert-butyl ether (MTBE) was added, and the mixture was shaken for 1 h. A subsequent step involved adding 250 μL of ddH2O and vortex-shaking for 1 min. The sample was then centrifuged (10 min, 12,000 rpm, 4°C), and 400 μL of the supernatant was collected. This liquid was concentrated using a vacuum concentrator until dry. Finally, 100 μL of isopropanol/acetonitrile (1/1) solution was added for sonic reconstitution for 1 min. The reconstituted sample was then filtered through a 0.22-μm membrane filter and prepared for analysis.

For spleen samples, one steel ball and 20 ceramic beads were added to each sample, which was then ground for 1 min. Subsequently, 300 μL of methanol and 1 mL of MTBE were added, and the mixture was shaken for 60 min to ensure thorough mixing. Afterward, the sample was centrifuged (10 min, 12,000 rpm, 4°C), and 300 μL of the supernatant was collected for concentration in a vacuum concentrator. A final step involved adding 100 μL of isopropanol/acetonitrile (1/1) solution for sonic reconstitution for 1 min. The reconstituted sample was filtered through a 0.22-μm membrane filter for analysis.

The samples were tested using the Thermo Scientific U3000 (Agilent Technologies, Santa Clara, CA, United States). Chromatographic separation was performed with an ACQUITY UPLC BEH C8 (2.1 mm × 100 mm, 1.7 µm, Waters, United States) and samples were maintained at 50°C. For the plasma samples, the mobile phase consisted of water/acetonitrile at 6/4, containing 0.1% formic acid and 5 mM ammonium formate (A), and isopropanol/acetonitrile at 1/9, containing 0.1% formic acid and 5 mM ammonium formate (B), in gradient elution at 0.3 mL/min. For ESI^+^, a linear gradient of 0–2 min, 0%–30% B; 2–9 min, 30%–70% B; 9–11 min, 70%–95% B; 11–12 min, 95%–100% B was employed. For ESI^−^, the linear gradient was 0–3 min, 10%–35% B; 3–6 min, 35%–85% B; 6–8 min, 85%–100% B. The injection volume and flow velocity were 1.0 μL and 0.3 mL/min, respectively.

The ion-source parameters were optimized and set as follows: capillary voltage, ESI^+^ 3.7 kV, ESI^−^ 3.5 kV; drying gas temperature, 320°C; drying gas flow rate, 10 L/min; nebulizer pressure, 30 psig; sheath gas temperature, 300°C; sheath gas flow rate, 12 L/min. Data were acquired at a rate of 1 spectrum/s in the scanning range of 150–1,500 m*/z*.

### 2.10 Statistical analysis

The chromatograms of 30 samples of water extract of RAS were input into the *Similarity Evaluation System for Chromatographic Fingerprint of TCMs* software package (Chinese Pharmacopoeia Commission, version 2012.130723) to generate the standard fingerprints of RAS of different grades. Chemometric analysis, including multivariate data analysis, was performed using the SIMCA 14.1 software package. One-way analysis of variance, followed by the Student’s t-test, was conducted to determine the statistical significance of differences between groups. The threshold for statistical significance was set at *p* < 0.05.

## 3 Results

### 3.1 High-performance liquid chromatography fingerprints and the nine main compounds in RAS

**FIGURE 1:**
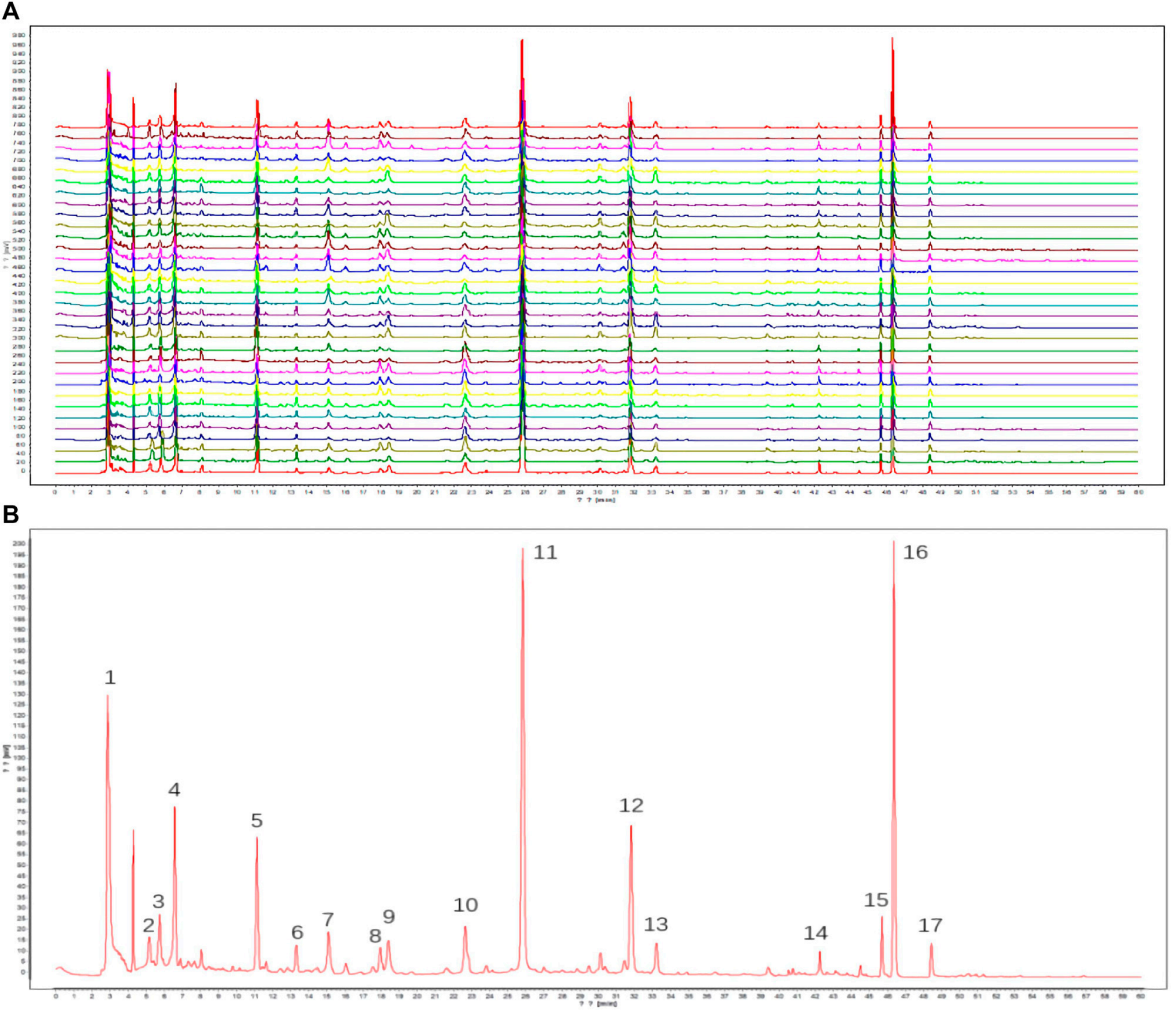
HPLC fingerprints of samples. (**(A)** Fingerprints of different grades of RAS; **(B)** Standard fingerprint of different grades of RAS. 1: unknown; 2: uridine; 3: adenosine; 4: guanine; 5: tryptophan; 6: unknown; 7: chlorogenic acid; 8: unknown; 9: unknown; 10: unknown; 11: ferulic acid; 12: senkyunolide I; 13: unknown; 14: unknown; 15: unknown; 16: ligustilide; 17: levistolide A).

The results of this methodological investigation are presented in Supplementary Table S1. All results indicated that the HPLC analysis method was valid and satisfactory. The HPLC profiles of 30 batches of RAS water extract are shown in Figure 1A. Seventeen coexisting peaks were detected in all analyzed samples; these were labeled 1–17 according to the standard fingerprint of RAS of different grades (Figure 1B). Indices of similarity among the samples of different grades were in the range of 0.871–0.997 (Table 1). These high degrees of similarity indicated that the compounds found in different grades of RAS were similar.

**TABLE 1:** Similarity of different grades of RAS.

Sample	Similarity	Sample	Similarity
1	0.979	16	0.997
2	0.988	17	0.871
3	0.985	18	0.991
4	0.993	19	0.954
5	0.983	20	0.983
6	0.994	21	0.986
7	0.982	22	0.981
8	0.973	23	0.996
9	0.990	24	0.994
10	0.969	25	0.979
11	0.969	26	0.981
12	0.994	27	0.991
13	0.933	28	0.992
14	0.978	29	0.982
15	0.980	30	0.963

Nine reference compounds were quantitatively analyzed: ferulic acid, ligustilide, senkyunolide I, chlorogenic acid, levistolide A, uridine, adenosine, guanine, and *L-*tryptophan. Among the four RAS grades (Table 2), the third group showed the highest average values for ferulic acid, ligustilide, senkyunolide I, levistolide A, uridine, adenosine, and guanine.

**TABLE 2:** The content (mg/g) of nine components in different grades of RAS.

Grade	No.	Ferulic acid	Ligustilide	Senkyunolide I	Chlorogenic acid	Levistolide A	Uridine	Adenosine	Guanine	*L*-tryptophan
First grade	1	9.97	27.20	1.85	2.57	12.78	19.00	96.27	54.65	17.24
	2	13.90	18.37	2.48	3.39	11.84	18.38	80.12	53.60	18.02
	3	9.22	20.83	0.88	1.83	12.58	14.13	71.65	38.20	11.88
	4	12.19	24.93	1.81	2.62	13.09	13.12	57.09	34.39	9.42
	5	8.26	15.96	0.95	3.43	12.46	21.14	86.53	51.98	13.38
	6	10.47	23.61	1.38	3.32	13.17	21.83	90.40	57.89	15.97
	7	13.25	21.41	2.94	2.63	12.97	14.94	60.22	42.57	3.98
	8	12.83	21.66	1.74	4.99	12.84	18.58	95.48	53.49	14.01
Second grade	9	17.40	23.57	1.92	3.09	11.04	15.18	62.50	41.40	11.70
	10	11.06	32.84	1.79	2.36	13.72	7.23	60.67	39.21	11.76
	11	14.25	19.59	2.45	4.13	12.43	15.92	82.70	53.08	9.79
	12	16.62	20.36	1.39	1.36	12.68	11.67	71.13	34.90	23.13
	13	6.52	16.98	0.87	3.41	12.97	11.54	67.11	39.48	10.79
	14	8.68	14.93	1.03	2.44	10.78	13.20	50.06	45.97	8.20
	15	12.78	21.04	1.34	3.47	13.48	15.84	85.54	42.70	14.32
	16	14.35	28.88	1.43	2.34	13.92	12.08	76.37	38.05	18.01
Third grade	17	15.50	31.56	3.58	3.71	13.09	20.93	82.93	51.92	18.02
	18	9.44	23.19	4.11	4.43	14.12	21.82	92.86	59.63	12.54
	19	13.36	34.74	1.43	2.91	14.18	20.46	111.55	58.18	14.54
	20	14.57	17.16	2.16	7.88	13.81	19.65	71.87	45.18	9.51
	21	13.85	29.20	2.90	4.71	13.42	19.19	94.86	54.75	13.70
	22	11.62	26.08	3.96	2.93	13.35	24.01	71.22	46.01	10.60
	23	11.05	21.98	4.64	6.79	15.07	13.34	81.86	37.77	12.56
Fourth grade	24	5.48	11.90	1.85	3.92	13.16	22.12	73.00	58.22	17.73
	25	13.74	20.27	1.64	7.03	13.46	13.47	81.79	46.06	12.79
	26	6.94	19.40	2.97	5.02	13.37	11.76	69.16	48.28	7.88
	27	8.98	16.07	2.37	9.01	13.24	11.69	54.19	42.11	10.79
	28	9.80	20.48	2.76	8.20	12.32	21.48	79.59	60.84	14.06
	29	10.80	21.07	1.14	3.47	13.97	14.64	99.98	44.04	12.23
	30	10.70	17.08	2.50	12.27	13.21	15.58	72.25	43.72	14.02

### 3.2 Anti-platelet aggregation activity

**FIGURE 2:**
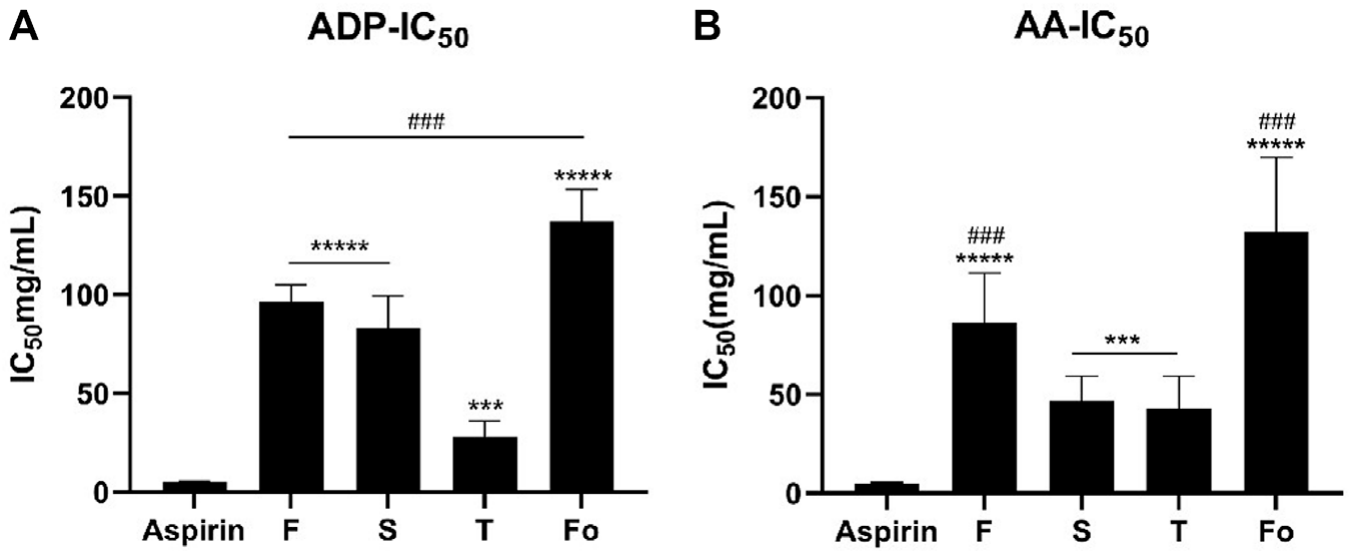
The IC50 of platelet aggregation activity induced by ADP or AA of 30 batches of RAS (**(A)** The IC50 of platelet aggregation activity induced by ADP; **(B)** The IC50 of platelet aggregation activity induced by AA). ***p < 0.001, *****p < 0.00001 vs Aspirin; ^###^ *p* < 0.001, ^#####^ *p* < 0.00001 vs the third-grade group.

The percentage of activities inhibiting platelet aggregation varied with RAS grade. As shown in Figure 2, illustrating the anti-platelet aggregation activity induced by ADP or AA of water extract samples with different grades of RAS *in vitro*, the ordering of different grades of RAS by IC_50_ was as follows: fourth-grade > first-grade > second-grade > third-grade (*p* < 0.001).

### 3.3 Spectrum–effect analysis

**FIGURE 3:**
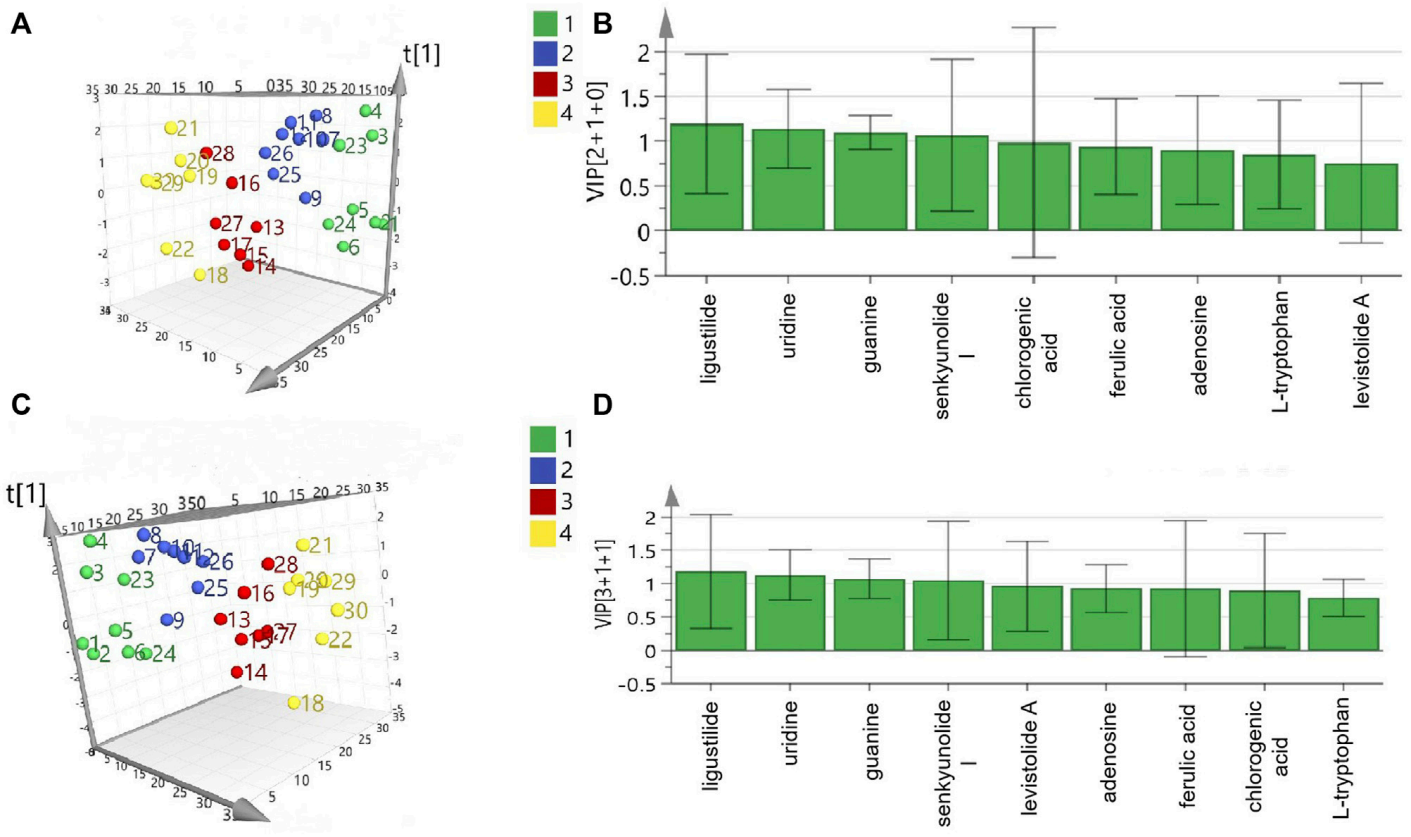
Result of PCA analysis of coexisting peaks and antiplatelet aggregation activities of different grades of RAS: adenosine diphosphate, PCA 3D plot **(A)** and VIP **(B)**; arachidonic acid, PCA 3D **(C)** and VIP **(D)**.

As shown in Table 3, nine main compounds were identified as bioactive compounds with anti-platelet aggregation activity through grey relational analysis. Levistolide A and senkyunolide I exerted the strongest influence on ADP- and AA-induced platelet aggregation activity, respectively. As shown in Figure 3A,C, the segregation produced via 3D principal component analysis (PCA) was between anti-platelet aggregation activity and the content of the different grades of RAS. Senkyunolide I, uridine, guanine, and ligustilide showed the greatest influence on ADP- and AA-induced platelet aggregation based on their variable importance in projection (VIP) scores (Figures 3B,D, respectively).

**TABLE 3:** Grey relational analysis and ranking results of nine bioactive components on ADP- and AA-induced anti-platelet aggregation activity.

Peak no.	Compound	γ_0, i_	
		ADP	AA
1	Uridine	0.6439	0.7121
2	Adenosine	0.6588	0.7037
3	Guanine	0.6825	0.7079
4	Tryptophan	0.6715	0.6838
5	Chlorogenic acid	0.6452	0.7104
6	Ferulic acid	0.6254	0.7007
7	Senkyunolide I	0.6127	0.7194
8	Ligustilide	0.6334	0.6929
9	Levistolide A	0.6915	0.7069

### 3.4 Body weight and spleen index

**FIGURE 4:**
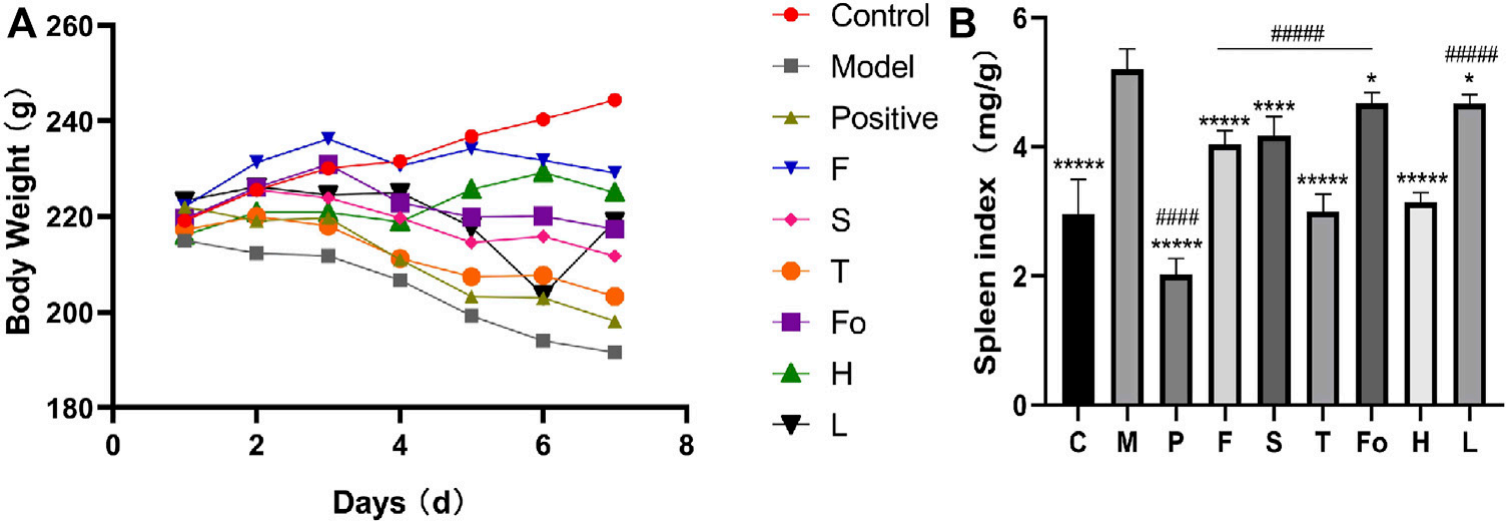
The body weight and the effect of RAS on spleen index in BD rats (**(A)** The body weight of rats; **(B)** The effect of RAS on spleen index in rats with blood deficiency. mean ± SD, n = 6) ****p < 0.0001, *****p < 0.00001 vs model group; ^####^ *p* < 0.0001, ^#####^ *p* < 0.00001 vs the third-grade group.

The functions of the spleen include hematogenesis, and the spleen plays a dominant role in blood control in TCM. The effects of BD on the spleen were evaluated by examining the spleen index. As shown in Figure 4A, the body weight of the model rats was significantly lower than that of the control rats. The RAS and positive control groups gained more body weight than the model rats. As shown in Figure 4B, the organ index for the spleen was significantly higher among the model group than the control group. The spleen index in the control group was 2.95 ± 0.55, while its value was 5.20 ± 0.32 in the model group. It was clear that the spleen index recovered to the normal level in the third-grade RAS dose group and the high-dose group, whereas it fell between the normal and model levels in the other RAS groups. All RAS groups were significantly different from the model group. It is worth mentioning that the aspirin group exhibited a decreased spleen index but also exhibited significantly decreased body weight, which indicated a lack of curative effect. These results suggest that water extract of RAS, especially third-grade RAS, could improve spleen enlargement in a rat model of BD.

### 3.5 Peripheral blood cells

**FIGURE 5:**
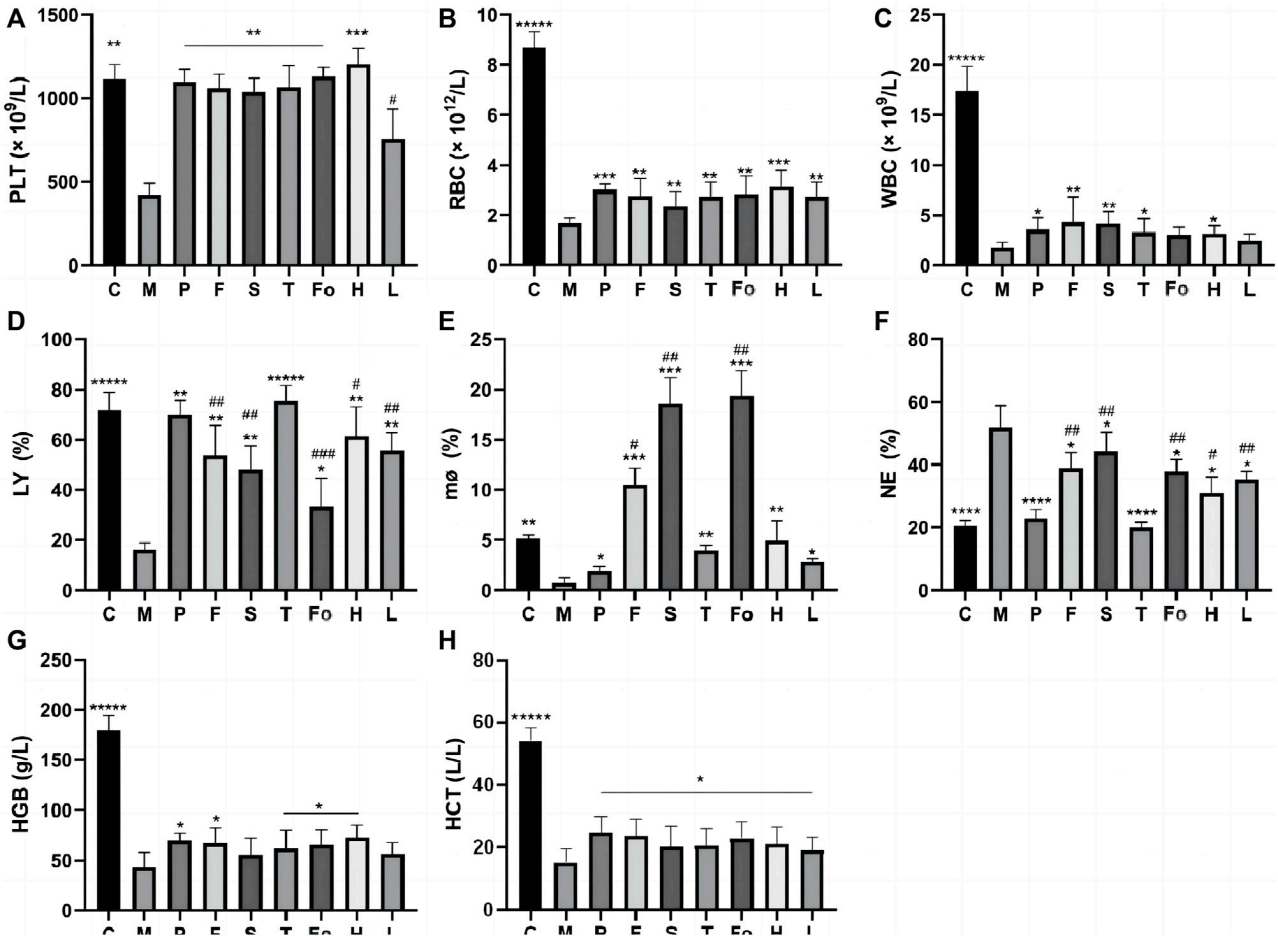
Effect of RAS on Peripheral Blood Cells in BD rats (mean ± SD, n = 6) *p < 0.05, ** *p* < 0.01, ***p < 0.001, *****p < 0.00001 vs model group; ^#^ *p* < 0.05, ^##^ *p* < 0.01 vs the third-grade group.

WBC, RBC, PLT, LY, MO, NE, HGB, and HCT are important indicators in routine blood examinations that can directly reflect the status of hematopoietic function in the rat model of BD. The results of these analyses are shown in Figures 5A–H. WBC, RBC, PLT, HGB, and HCT levels were significantly lower in the model group than in the control group (*p* < 0.0001), indicating that the BD model was successfully replicated. After treatment with RAS, these levels increased to varying degrees (*p* < 0.01), and the PLT index recovered to normal levels (*p* < 0.01), which is consistent with anti-platelet aggregation activity *in vitro*. Notably, levels of LY, MO, and NE in the third-grade RAS group were similar to those in the control group, and there were significant differences between the third-grade group and the other RAS groups.

### 3.6 Pathological morphology of the heart, kidney, and spleen

**FIGURE 6:**
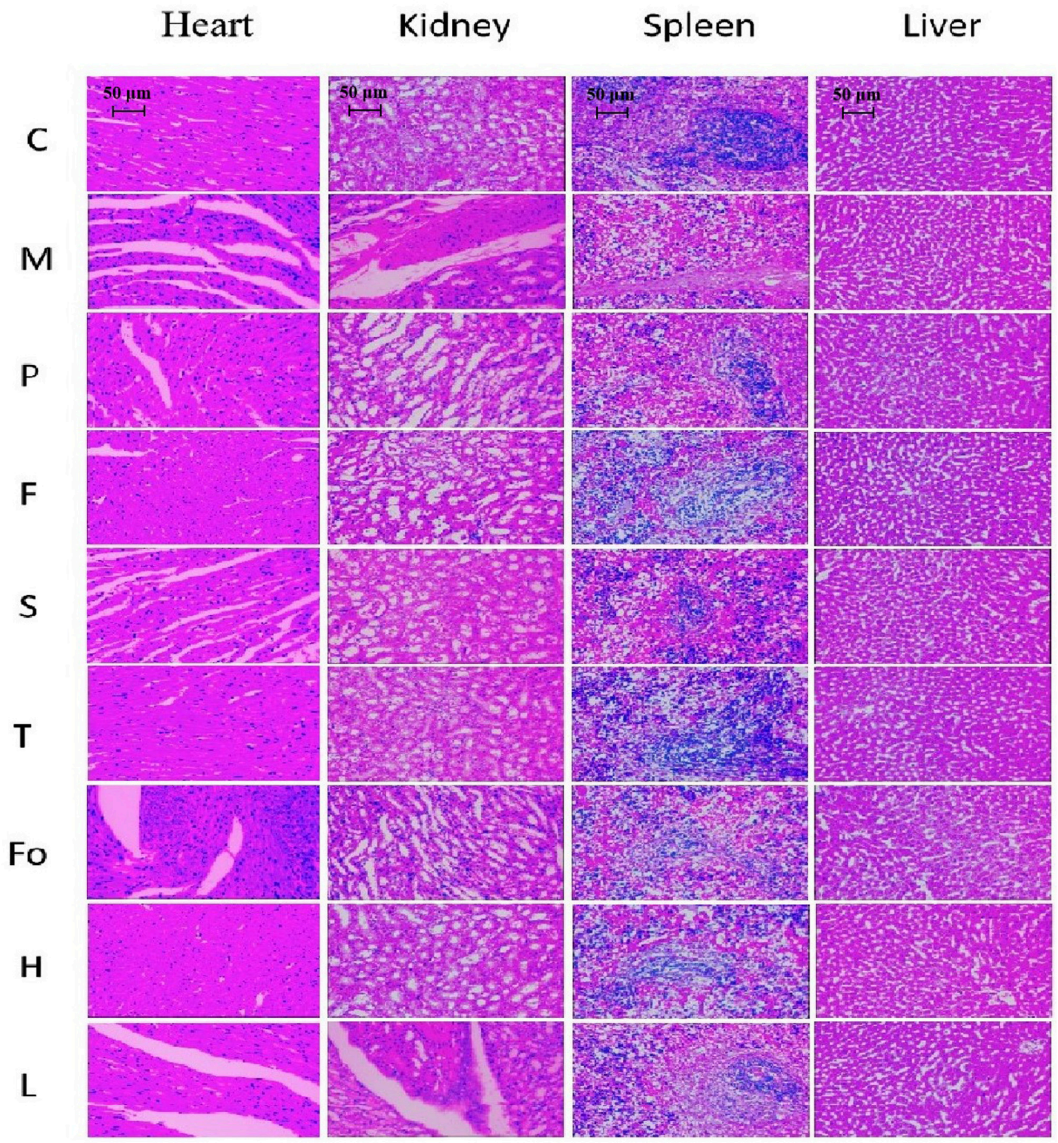
Effect of RAS on the heart, kidney, spleen, and liver in BD rats (H & E staining, × 200).

HE staining results for each group are shown in Figure 6. Differences in pathological morphology slices between the model and control groups were clearly observed for the heart, kidney, and spleen. However, there were no clear abnormalities in the liver slices. The heart endometrium, the myocardium, the epicardial structure, and horizontal stripes of myocardial cells were clearly observed in the control group, and the myocardial fibers were evenly colored. The model group showed significant changes in neutrophilic granulocyte augmentation and interstitial edema. The control group exhibited normal renal morphology without pathological lesions; however, vacuoles and focal segmental sclerosis were observed in the glomeruli of the model group. In the spleen slices, the purplish-red part represents the red pulp; the white pulp, formed by the aggregation of lymphocytes, was dyed blue. The proportion of white pulp was significantly reduced in the model group. There was significant recovery from damage in the other groups, whether they were treated with RAS or with aspirin. Specifically, the effect in rats in the RAS groups, with the exceptions of the fourth-grade and low-dose third-grade groups, was better than that observed in the positive control group. Moreover, the spleen was significantly larger in the model group than in the other groups. After treatment with RAS and aspirin, the spleen decreased in size. The third-grade RAS group exhibited excellent therapeutic effects on lesions of the heart, kidney, and spleen. These findings were similar to the results on anti-platelet aggregation activity *in vitro*.

### 3.7 Activity of ATPase in plasma

**FIGURE 7:**
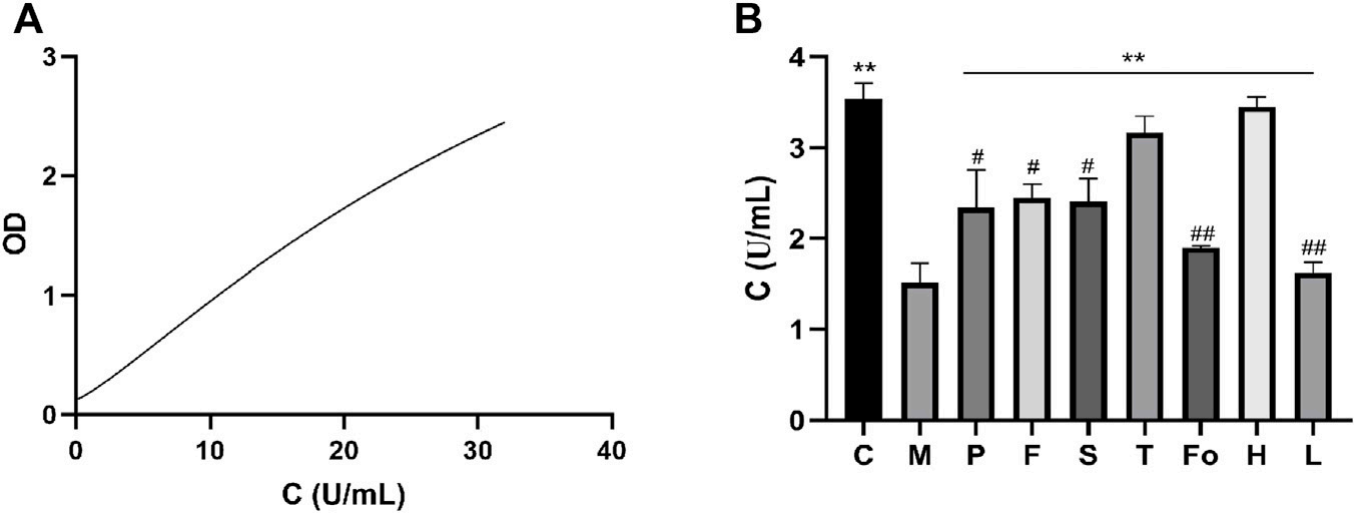
Effect of RAS on the content of ATPase activity in BD rats (mean ± SD, n = 3). **p < 0.01, vs model group; #*p* < 0.05, ##*p* < 0.01, vs the third-grade group.

The standard enzyme-linked immunosorbent assay curve is shown in Figure 7A. ATPase content was 3.54 ± 0.17 U/mL for the control group and 1.51 ± 0.22 U/mL for the model group. Relative to the control group, the ATPase content observed in the model group was significantly decreased (*p* < 0.01). In contrast, ATPase content was increased in the RAS and aspirin groups. In particular, ATPase content in the third-grade group increased to the level of the control group, indicating that the third-grade group exhibited a superior effect in terms of the development of ATPase content (Figure 7B).

### 3.8 Multivariate statistical analysis of metabolomics

**FIGURE 8:**
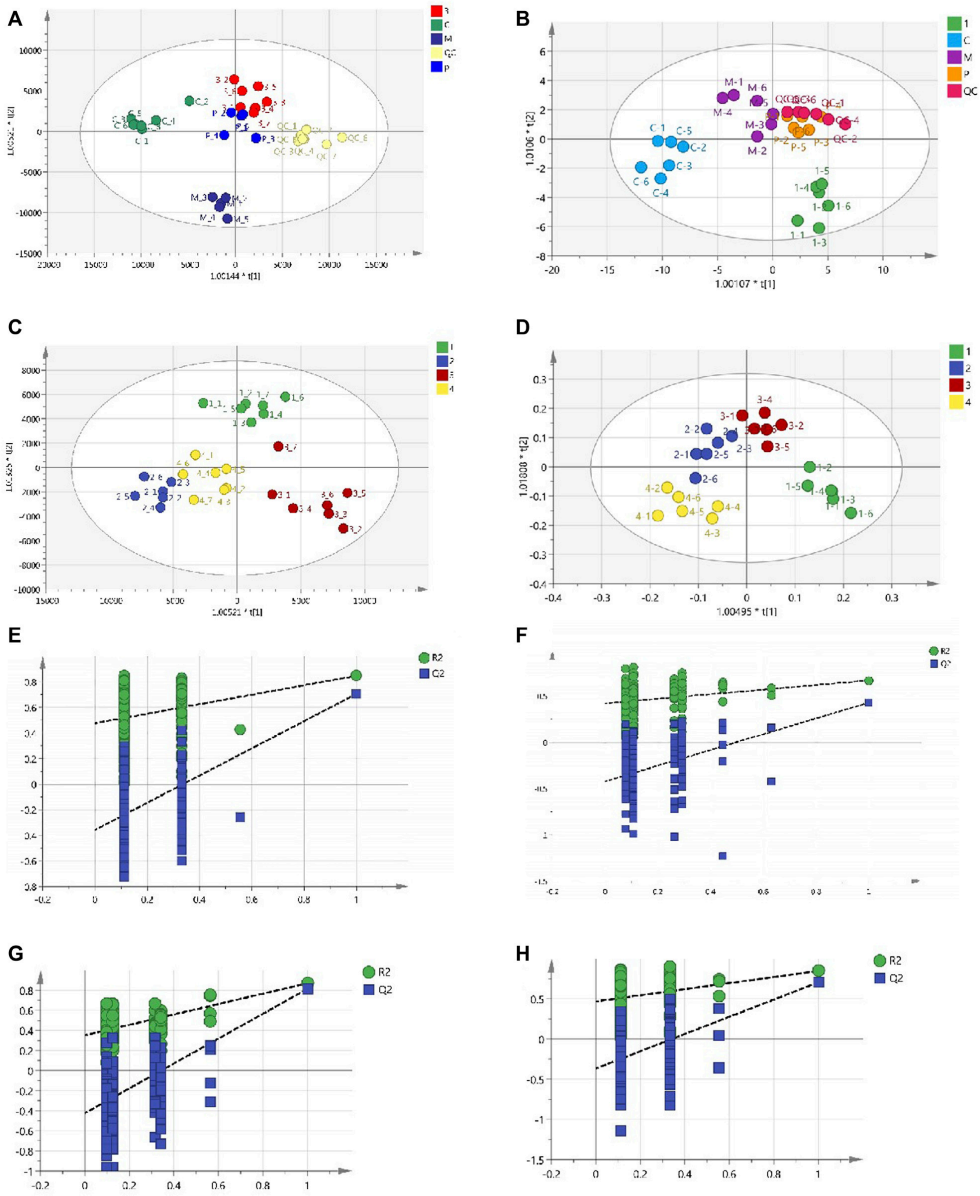
OPLS-DA score plot and ermutation test plot of the OPLS-DA models. (**(A)** OPLS-DA score plot of the control group, model group, positive group, the third-grade group of RAS, and QC group in plasma; **(B)** OPLS-DA score plot of the control group, model group, positive group, the first-grade group of RAS, and QC group in urine; **(C)** OPLS-DA score plot of different grade groups of RAS in plasma; **(D)** OPLS-DA score plot of different grade groups of RAS in urine; **(E)** The control group, model group, positive group, the third-grade group of RAS, and QC group samples of 200 permutation times in plasma; **(F)** The different grade samples of 200 permutation times in plasma; **(G)** The control group, model group, positive group, the first-grade group of RAS, and QC group samples of 200 permutation times in urine; **(H)** The different grade samples of 200 permutation times in urine).

Total ion chromatograms (TICs) of plasma and urine from different groups in positive mode are shown in Supplementary Figure S2. Endogenous metabolites in the plasma and urine of the BD model rats were selected using VIP scores. The differential endogenous metabolites of plasma and urine, screened for VIP >1.5 and *p* < 0.05, along with information on their identification, are presented in Tables 4, 5, respectively. The peak area and retention time of ions, determined using Profinder B.08.00, were imported into the SIMCA 14.1 software package. Permutation plots of plasma in positive mode were constructed to verify the reliability of the multidimensional statistical model, and the permutation test showed that the model was not overfitting. All plasma and urine samples were subjected to OPLS-DA based on the differential metabolites identified. A summary of the various chemometric models used in the analysis of the UPLC-QTOF-mass spectra is provided in Supplementary Table S2. As shown in Figures 8A,B, OPLS-DA score plots of plasma and urine in the control, model, aspirin, third-grade RAS, and QC groups in positive mode were clearly separated, indicating that blood deficiency occurred after injection with APH and CTX, and that RAS was able to improve the metabolic disturbance induced by BD. Clear separation between the first-to fourth-grade groups can be observed in Figures 8C,D, showing that different influences on plasma and urine were in effect under each grade of RAS. The constructed model fit the obtained data well, with Q^2^Y suggesting that the model could accurately predict new data for plasma and urine according to a permutation test with 200 repetitions, as shown in Figures 8E–H.

**TABLE 4:** Information on the identification of differential endogenous metabolites in the plasma of rats with blood deficiency (trend: model vs. control group).

No.	T_R_ (time)	Observed	VIP	Molecular formula	Metabolites	MS/MS fragment ion (m/z)	Trend
		m/z (ESI+)					
1	11.28	560.3367 [M + FA + Li] ^+^	10.66	C_25_H_52_NO_7_P	LysoPC(17:0) (Wang et al., 2022)	86.0963, 104.1069, 184.0732, 500.2446, 524.3130, 542.3235	↑
2	10.82	818.5822 [M + Na]^+^	6.33	C_45_H_83_N_2_O_7_P	SM(d20:1/20:4 (5Z,7E,11Z,14Z)-OH(9))	337.2517, 355.2627, 373.2733, 799.5704	↑
3	20.45	759.5700 [M + H]^+^	5.41	C_42_H_80_NO_8_P	PC(18:1 (9Z)-O (12,13)/P-16:0)	184.0732	↑
4	15.37	546.3528 [M + Na]^+^	4.45	C_26_H_54_NO_7_P	LysoPC(0:0/18:0)	86.0946, 91.0535, 184.0655, 238.0989, 261.1334	↑
5	9.54	305.2983 [M + H]^+^	4.4	C_20_H_32_O_2_	Arachidonic acid (Bian et al., 2023)	44.9971	↑
6	15.48	611.4128 [M + Na]^+^	4.18	C_39_H_56_O_4_	Caffeoylcycloartenol	89.0608, 207.0626, 269.2481	↓
7	15.31	613.4323 [M + CH_3_OH + H]^+^	3.47	C_33_H_56_O_8_	DG (PGE2/0:0/10:0)	46.0655, 287.2359, 331.2636	↓
8	10.45	464.2823 [M + H]^+^	3.29	C_22_H_23_O_11_	LysoPA (20:2 (11Z,14Z)/0:0)	97.9759,105.0332, 207.0816, 311.1207, 405.2041	↑
9	11.23	393.5709 [M + H]+	3.06	C_24_H_40_O_4_	Chenodeoxycholic acid (Zhan et al., 2012)	82.0012, 128.1031	↓
10	8.98	162.1985 [M + H]^+^	2.92	C_7_H_15_NO_3_	*L*-Carnitine (Wu et al., 2016)	74.0486, 89.0711	↓
11	14.78	759.5778 [M + H]^+^	2.04	C_42_H_80_NO_8_P	PE (15:0/22:2 (13Z,16Z))	86.0964, 184.0730	↑
12	7.16	301.1412 [M + Na]^+^	1.78	C_16_H_22_O_4_	Diisobutyl phthalate	45.0338, 67.0544, 89.0602	↑
13	1.06	90.0555 [M + H]^+^	1.73	C_3_H_7_NO_2_	*L*-Alanine (Huang et al., 2018)	116.0936	↑
14	11.03	637.5191 [M + H]^+^	1.73	C_41_H_81_NO_3_	Ceramide (Li et al., 2022)	74.0459, 312.2797, 398.3083	↑
15	6.32	176.0951 [M + H]^+^	1.66	C_6_H_13_N_3_O_3_	Citrulline (Zhang, 2020)	143.7347, 156.9812	↓
16	8.53	129.0658 [M + H]^+^	1.65	C_5_H_8_N_2_O_2_	Dihydrothymine	76.1312	↓
17	1.74	132.1735 [M + H]^+^	1.64	C_6_H_13_NO_2_	*L*-Leucine (Zhu et al., 2020)	102.1044	↓
18	3.61	147.0773 [M + H]^+^	1.63	C_5_H_10_N_2_O_3_	*L*-Glutamine (Yang et al., 2013; Wang et al., 2015)	155.4121	↓
19	15.59	482.2997 [M + H]^+^	1.52	C_28_H_37_N_2_O_5_	Unknown	325.2018, 461.1941	↑

**TABLE 5:** Information on the identification of differential endogenous metabolites in the urine of rats with blood deficiency (trend: model vs. control group).

No.	T_R_ (time)	Adduct	VIP	Molecular formula	Metabolites	MS/MS fragment ion (m/z)	Trend
		*m/z* (ESI^+^)					
1	13.16	751.5080 [M + Na]^+^	4.39	C_41_H_76_O_8_P	Cardiolipins (CL)	329.2477, 603.5331, 738.5012	↓
2	0.66	369.4645 [M + H]^+^	1.91	C_20_H_32_O_6_	Endoperoxide G2	295.1094	↑
3	7.34	315.2060 [M + H]^+^	1.89	C_19_H_26_N_2_O_2_	Unknown	286.1544	↓
4	6.71	289.2260 [M + Li]^+^	1.79	C_19_H_26_N_2_	Aspidospermidine (Feng, 2019)	41.0386, 255.1809, 267.1809	↓
5	8.32	338.1875 [M + K]^+^	1.78	C_20_H_29_NO	Unknown	83.0851	↑
6	8.30	132.1019 [M + H]^+^	1.72	C_6_H_13_NO_2_	*L*-lsoleucine	86.0979, 91.0541	↓
7	5.32	180.1727 [M + H]^+^	1.61	C_9_H_9_NO_3_	hippuric acid (Yang, 2019)	91.1137, 119.0007	↓
8	1.21	819.4525 [M + H]^+^	1.61	C_41_H_72_NO_11_PS	PA (LTE4/*i-*15:0)	189.0935, 391.2663	↑
9	1.07	216.1233 [M + Na + H]^+^	1.59	C_6_H_8_O_7_	Citric acid (Huang et al., 2020)	133.0137	↓
10	4.93	863.6179 [M + H]^+^	1.57	C_46_H_87_NO_13_	Galabiosylceramide (Ferraris et al., 2022)	145.0501,163.0598, 708.5770	↓
11	7.19	234.0516 [M+2Na] ^+^	1.54	C_7_H_12_N_2_O_4_	Glycylhydroxyproline	44.9965	↓
12	13.14	795.9741 [M + Li]^+^	1.52	C_42_H_77_O_11_P	PA (*i-*19:0/PGE1)	96.9685	↑

**FIGURE 9:**
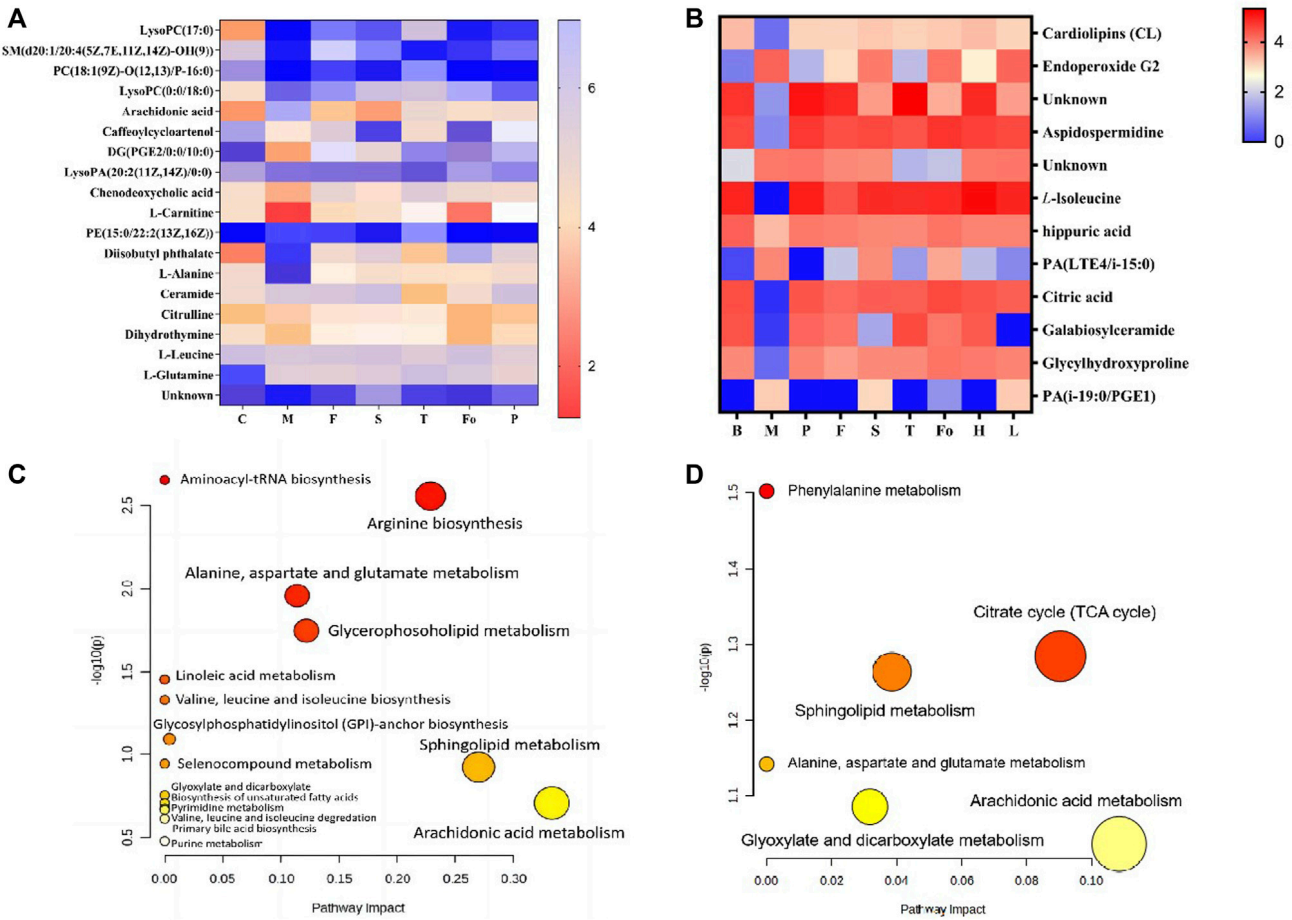
The heatmap visualization of the differential metabolites in plasma **(A)** and urine **(B)**, altered metabolomic pathways observed in plasma sample after RAS treatment in plasma **(C)** and urine **(D)**.

Based on their VIP scores, *p*-values, and S-plot results, 19 potential endogenous metabolites (Table 4) in plasma were screened and identified, based on MS/MS fragments, retention behavior, and online databases such as Metlin (http://metlin.scripps.edu/), LIPID MAPS (http://www.lipidmaps.org), and the Human Metabolome Database (HMDB, http://www.hmdb.ca), as differential metabolites associated with the rat model of BD. Compared with the control group, 11 metabolites were significantly upregulated (*p* < 0.01) in the model group, including lysophosphatidylcholine LysoPC(17:0), sphingomyelin SM(d20:1/20:4 (5Z,7E,11Z, 14Z)-OH(9)), phosphatidylcholine PC(18:1 (9Z)-O (12,13)/P-16:0), LysoPC(0:0/18:0), arachidonic acid, LysoPA (20:2 (11Z, 14Z)/0:0), phosphatidyl ethanolamine PE (15:0/22:2 (13Z, 16Z)), diisobutyl phthalate, *L*-alanine, and ceramide. In contrast, the levels of eight metabolites were clearly downregulated (*p* < 0.01): these were caffeoylcycloartenol, DG (PGE2/0:0/10:0), chenodeoxycholic acid, *L-*carnitine, citrulline, dihydrothymine, *L-*leucine, and *L-*glutamine. Twelve differential metabolites were detected in urine samples. Four metabolites were upregulated in the model group, including endoperoxide G2, PA (LTE4/*i-*15:0), and PA (*i-*19:0/PGE1); and eight metabolites were downregulated in the model group, including hippuric acid, citric acid, and galabiosylceramide. Figures 9A,B show heatmap images of the differential metabolites in plasma and urine, respectively. After treatment with RAS, the metabolites showed different degrees of increase or decrease in the process of recovery to control levels. The MetaboAnalyst 4.0 (https://www.metaboanalyst.ca/MetaboAnalyst/home.xhtml) system was used to analyze the pathways of plasma and urine metabolites in the rat model of BD. As shown in Figure 9C, the important pathways of plasma metabolites in relation to RAS treatment in the rat model of BD included arachidonic acid metabolism; arginine biosynthesis; alanine, aspartate, and glutamate metabolism; sphingolipid metabolism; and glycerophospholipid metabolism. Four main metabolic pathways were identified in urine: the tricarboxylic acid (TCA) cycle, sphingolipid metabolism, arachidonic acid metabolism, and glyoxylate and dicarboxylate metabolism (Figure 9D).

### 3.9 Multivariate statistical analysis of lipidomics

**FIGURE 10:**
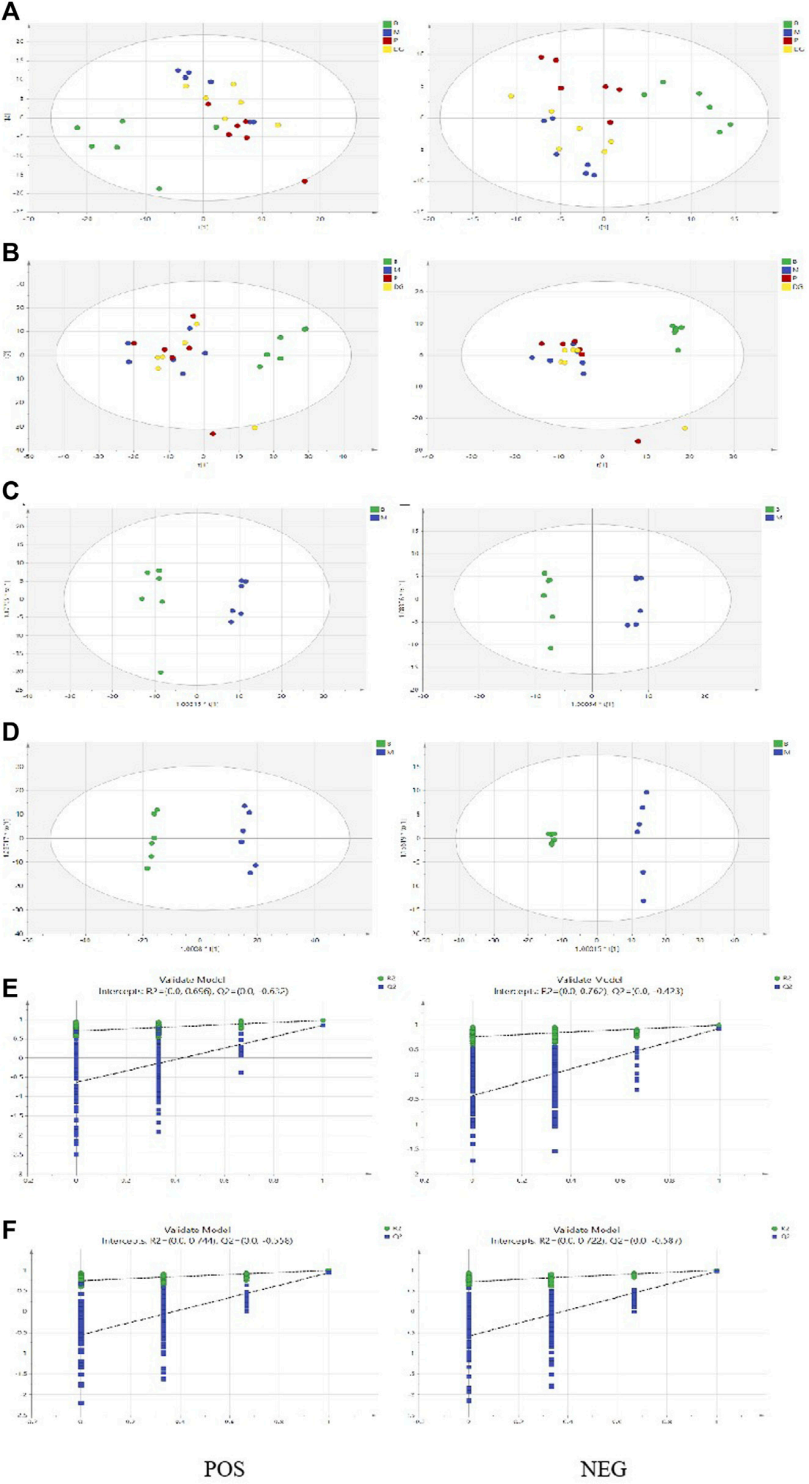
PCA score plot, OPLSDA score plot, and ermutation test plot of the OPLS-DA models. (**(A)** PCA score plot of the control group, model group, positive group, and the third-grade group of RAS in plasma via positive and negative model; **(B)** PCA score plot of the control group, model group, positive group, and the third-grade group of RAS in urine via positive and negative model; **(C)** OPLS-DA score plot of the control group and model group in plasma via positive and negative model; **(D)** OPLS-DA score plot of the control group and model group in urine via positive and negative model; **(E)** The control group and model group samples of 200 permutation times in plasma via positive and negative model; **(F)** The control group and model group samples of 200 permutation times in urine via positive and negative model).

TICs in the plasma and spleen are shown in Supplementary Figure S3. All groups of plasma and spleen were re-analyzed via PCA and orthogonal projections to latent structures discriminant analysis (OPLS-DA) using the SIMCA 14.1 software package. A summary of the various chemometric models used in the analysis of the UPLC-Q-exactive-mass spectra is shown in Supplementary Table S3. As shown in Figures 10A,B, PCA score plots for the plasma and spleen in the positive and negative modes showed clear separation in the control, model, aspirin, and third-grade RAS groups. OPLS-DA score plots for the plasma and spleen in positive and negative modes in the control and model groups are shown in Figures 10C,D. The constructed model fit the obtained data well, with Q^2^Y suggesting that the model could accurately predict new data based on 200 permutation tests, as shown in Figures 10E,F. Endogenous metabolites in the plasma and spleen of the BD model rats were selected using VIP scores. The differential endogenous metabolites of plasma and spleen, screened for VIP >1.5 and *p* < 0.05, are shown in Supplementary Tables S4, S5, respectively.

**FIGURE 11:**
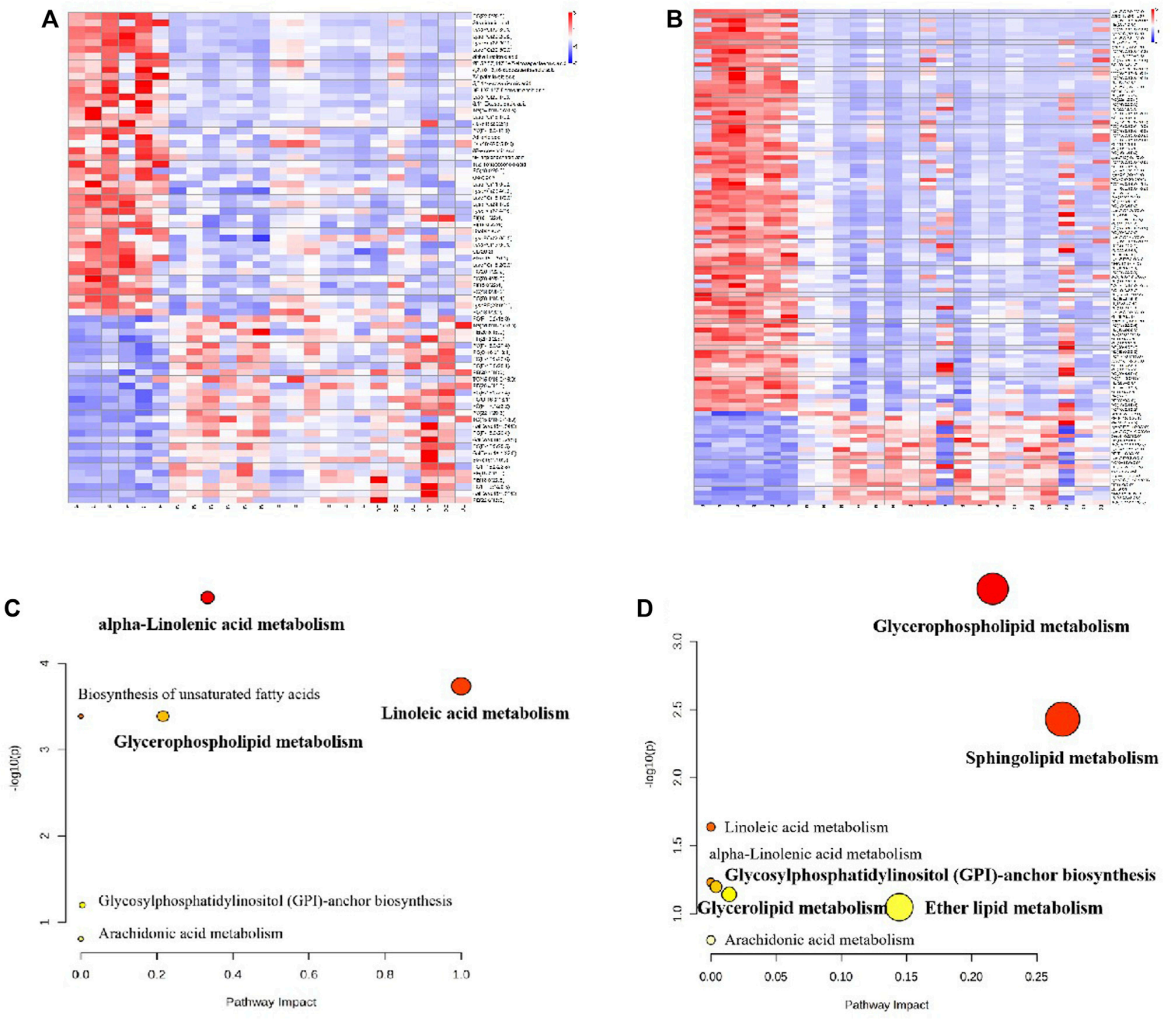
The heatmap visualization of the differential lipid metabolites in plasma **(A)** and urine **(B)**, altered lipid metabolomic pathways observed in plasma sample after RAS treatment in plasma **(C)** and urine **(D)**.

Approximately 73 potential biomarkers were screened in plasma. Polyunsaturated fatty acids and glycerophospholipids were the two most clearly evident changes observed in the plasma lipidomics of BD rats. Twenty-eight metabolites were upregulated in plasma in the model group, including PC (P-18:0/18:3), triglyceride TG (16:0/16:0/16:0), and PE (20:5/18:0); and 45 metabolites were downregulated, including PC (22:2/20:5), α-linolenic acid, and linoleic acid. Each RAS group exhibited regulation of 73 potential biomarkers. A total of 112 potential biomarkers were identified in the spleen. The two most prominent substances were TG. Twenty-one metabolites, including PE (20:4/22:2), PC (P-18:0/22:5), and LysoPC (P-18:0/0:0), were upregulated in the spleen in the model group, and 91 metabolites were downregulated, including LysoPC (20:2/0:0), TG (14:0/16:0/20:4), and PE (20:1/0:0). These 112 potential biomarkers were upregulated in each treatment group. Figures 11A,B show heatmap images of the differential metabolites in the plasma and spleen, respectively. As shown in Figure 11C, three main metabolic pathways for plasma were screened for with MetPA: α-linolenic acid metabolism, glycerophospholipid metabolism, and linoleic acid metabolism. Five major metabolic pathways in the spleen were identified: glycerophospholipid metabolism, sphingolipid metabolism, glycerolipid metabolism, glycosylphosphatidylinositol-anchor biosynthesis, and ether lipid metabolism (Figure 11D).

## 4 Discussion

The use of RAS for enhancing blood circulation has a long history in TCM. The commercial grade specification for TCM is crucial in ensuring high-quality and premium price TCM materials. Distinguishing between commodity grades in terms of the differences in quality is essential, as it helps to improve commodity standards.

In the current study, we employed HPLC fingerprints and *in vitro* anti-platelet aggregation assays to identify the active components within different grades of RAS. Grey correlation analysis revealed that nine components exhibited inhibitory effects on ADP-induced and AA-induced platelet aggregation. Notably, there was a consistent correlation between anti-platelet aggregation activity and the content of different grades of RAS. Specifically, third-grade RAS demonstrated the most significant potential for enhancing blood circulation, particularly with regard to ADP- and AA-induced platelet aggregation activity. Conversely, fourth-grade RAS was associated with lower content of these components, mirroring its reduced anti-platelet aggregation activity. First- and second-grade RAS exhibited moderate effects on anti-platelet aggregation activity and fell in the middle range concerning the associated content of the relevant components among the various grades. Senkyunolide I, uridine, guanine, and ligustilide emerged as pivotal components for enhancing blood circulation in ADP- and AA-induced platelet aggregation. This corroborates our earlier findings, which identified senkyunolide I, uridine, guanine, ferulic acid, and adenosine as potential Q-markers for RAS (Zhang et al., 2022b). Additionally, our previous research identified ferulic acid, ligustilide, and senkyunolide I as Q-markers for anti-platelet aggregation and Ca^2+^ antagonistic activities using near-infrared reflectance spectroscopy (Gao et al., 2022). In other studies, ferulic acid, ligustilide, and levistilide A have also been identified as Q-markers for RAS through effective component-bioactivity network analysis (Liu et al., 2021). Furthermore, ferulic acid, levistilide I, and levistilide A have been found to alleviate RBC aggregation (Hong et al., 2003). In this study, we selected ferulic acid, ligustilide, senkyunolide I, uridine, and guanine as Q-markers of RAS to assess their impact on blood circulation.

In a rat model of BD, APH and CTX were administered to assess the efficacy of TCM in enhancing blood circulation (Lu, 2015). APH, known for its potent oxidizing properties, inflicts oxidative damage on RBCs, leading to hemolytic anemia within the organism (Roth et al., 1979). Conversely, CTX serves as a cytotoxic agent that targets bone marrow cells. The destruction of these bone marrow cells prompts the spleen (Pi) to assume responsibility for immune hematopoiesis, resulting in enlargement of the spleen (Wang et al., 2009). However, this enlargement of the spleen can lead to hypersplenism and impairment of immune functions. In TCM, the spleen is regarded as crucial in managing blood and enhancing blood circulation. In modern medical science, the spleen is recognized as a vital central immune organ, housing cytotoxic T lymphocytes and delayed-type hypersensitive T lymphocytes (Yao et al., 2021). RAS has been found to elevate PLT levels, restore spleen indices, alleviate organ damage, and enhance ATPase activity in erythrocyte membranes. In this study, histopathological results revealed an increase in neutrophilic granulocytes and interstitial edema in the heart, vacuoles and focal segmental sclerosis in the glomerulus, and a significant reduction in the white pulp in the spleen of the model group. Different grades of RAS exhibited varying degrees of efficacy in promoting blood circulation. Notably, third-grade RAS demonstrated the most favorable activity, aligning with the findings of the compound content analyses and *in vivo* and *in vitro* experiments.

Metabolomics and lipidomics analyses were conducted on BD rats, focusing on glycerophospholipids, amino acids, and polyunsaturated fatty acids (PUFAs), among other components, in comparison to the model group. Following treatment with RAS, the levels of metabolites and lipids were restored to normal. Notably, RAS was found to regulate the levels of certain glycerophospholipids in BD rats. Glycerophospholipids, including LysoPCs and PCs, are the most abundant phospholipids in the endoplasmic reticulum. They play crucial roles in cell membrane protein recognition, signal transduction, maintaining the body’s energy metabolism, and promoting material transport. LysoPCs, a subset of lysoglycerophospholipids, are particularly abundant in the blood, especially in plasma. When combined with a protein carrier, LysoPC plays a key role in facilitating the transfer of fatty acids for the growth and development of the brain and the body. It also serves as a fundamental component for synthesizing nerve cell membranes (Han et al., 2016). In an extracellular context, elevated concentrations of LysoPC can disrupt cell membrane integrity, leading to membrane rupture and subsequent toxicity (Ma et al., 2019). LysoPC is a significant pharmacodynamic marker associated with RAS efficacy in BD rats. It activates transient receptor potential 6 (TRPC6) channels, which in turn trigger the lipid-cleaving enzyme phospholipase A2 (PLA2) to release AA from cellular membranes, allowing calcium influx (Putta et al., 2021). The activation of the arachidonate lipoxygenase-15/12-hydroxyeicosatetraenoic acid (Alox15/12-HETE) signaling pathway during AA lipoxygenase metabolism serves to protect calcified blood vessels (Han et al., 2021). Furthermore, previous research has demonstrated that RAS obtained from geoherbs exhibits robust calcium-antagonistic activity (Zhang et al., 2019a).

LysoPC is recognized as a critical proinflammatory cytokine in atherosclerosis (AS), impacting endothelium-dependent vasorelaxation and vascular smooth muscle cells, and influencing platelet aggregation and coagulation pathways (Matsumoto et al., 2007). Importantly, LysoPCs possess hypoglycemic and anti-inflammatory properties. PCs and PEs, primarily found in cell membranes and lipoproteins, constitute over 50% of glycerophospholipids. PC, in particular, can inhibit inflammation (Guzzolino et al., 2018), influence macrophage phagocytosis strength, regulate immunity, and impact organelle biosynthesis, secretion, and endocytosis (Sasset et al., 2016). Simultaneously, PC contributes to the removal of peroxides from the plasma, breaks down cholesterol and fatty acids, reduces cholesterol and neutral fat levels in the blood, accelerates the excretion of fat, shortens its retention time, and restores the elasticity of blood vessel walls (Snider et al., 2018). In contrast, the accumulation of SM in whole blood increases viscosity and is considered a risk factor for coronary heart disease (Sasset et al., 2016). The reduction in SM levels indicates that RAS may decrease whole-blood viscosity, potentially preventing cardiovascular disease.

Furthermore, L-glutamine exerts its influence by inhibiting the production of reactive oxygen species, specifically oxygen-free radicals. It accomplishes this by activating the nuclear factor erythroid-2 related factor 2/antioxidant response element (Nrf2/ARE) signaling pathway, which in turn regulates levels of superoxide dismutase and glutathione (Xue et al., 2018). In the present study, L-glutamine and citrulline, both critical for arginine synthesis, were found to be downregulated in the model group. This downregulation results in significant inhibition of mitochondrial biosynthesis and ATP production (Hai, 2009). Furthermore, excessive pyruvate and other metabolites, along with disruptions in arginine and proline metabolism, interferes with the TCA cycle (Wang et al., 2013), ultimately reducing hemoglobin production (Hao and Yan, 2015). Additionally, an excess of ceramide accumulation was noted, causing disturbances in sphingolipid metabolism. Sphingolipids are implicated in potential mechanisms underlying cardiovascular disease (Jia et al., 2022). RAS can rectify sphingolipid metabolism by downregulating ceramide from a disordered state to normal levels. The breakdown of lipids into acetyl-CoA also impacts the TCA cycle and ATP production (Hua et al., 2017). Notably, nineteen potential endogenous metabolites primarily participated in five metabolic pathways. RAS has been demonstrated to enhance blood circulation by modulating the metabolism of arachidonic acid; arginine biosynthesis; alanine, aspartate, and glutamate metabolism; sphingolipid metabolism; and glycerophospholipid metabolism. Managing energy metabolism is considered to be a fundamental strategy in preventing hemolysis and treating BD (Zhang et al., 2022a). This approach involves regulating the osmotic pressure of blood cells and bolstering their synthesis. In summary, RAS effectively reduces the accumulation of AA, thereby shortening the period of activity of TRPC6 channels, enhancing calcium antagonistic activity, and participating in five key metabolic pathways.

BD model rats have been reported to exhibit metabolic disturbances in their blood. RAS has been shown to exert regulatory effects on various metabolic pathways, including the ornithine cycle, platelet biochemical pathways, fructose metabolism, lipid metabolism, the folate cycle, and energy metabolism (Hua et al., 2017). Furthermore, Angelica sinensis polysaccharides (ASP) and phthalides have demonstrated significant impact on the BD model. ASP, for instance, inhibits Wnt/β-catenin signaling pathway-mediated premature senescence and oxidative stress in hematopoietic cells, thereby mitigating oxidative damage and regulating perivascular niche function (Niu et al., 2023). Additionally, ASP protects hematopoietic stem/progenitor cells and prevents hematopoietic regression by enhancing the hematopoietic microenvironment (Jing et al., 2023). Ligustilide, a compound with a crucial role in enhancing blood circulation, offers protection against nerve damage caused by ischemic stroke. It accomplishes this by modulating the Drp1-mediated mitochondrial fission pathway by activating AMP-activated protein kinase (AMPK) signaling (Wu et al., 2022b). Ligustilide also safeguards vascular endothelial cells from oxidative stress by acting as an effective activator of Nrf2 (Liang et al., 2021). Ferulic acid can alleviate atherosclerotic plaques by inhibiting the proliferation of vascular smooth muscle cells (Wu et al., 2022a). Additionally, ferulic acid can impede the synthesis of triglycerides and cholesterol by upregulating adenosine monophosphate or ATP, which is sensed by AMPK (Gao et al., 2022). Finally, senkyunolide I has been shown to protect the brain from focal cerebral ischemia-reperfusion injury by binding to a promoter known as ARE, leading to the upregulation of several antioxidant and detoxification genes (Hu et al., 2015).

Urine constitutes a significant component in the body’s metabolic processes, and investigating differences in metabolites holds profound importance in comprehending the overall integrity of these processes. Citric acid, a vital precursor for the synthesis of essential fatty acids and a source of inflammatory mediators like prostaglandin and nitric oxide (Hu et al., 2015), plays a crucial role in metabolism. Amino acid metabolism is a carbon source for the energy cycle (Hao and Yan, 2015). Glutamine can be enzymatically broken down by glutaminase into glutamic acid and α-ketoglutaric acid, which in turn provide energy for the TCA cycle (Hao and Yan, 2015). The TCA cycle, a convergence point for the catabolism of sugars, fats, and amino acids, draws on intermediates from various biosynthetic pathways (Hua, 2014). Consequently, changes in the TCA cycle may reflect shifts in overall energy metabolism. Observing the citric acid content in the model group in the present study, we noted a decrease followed by an increase after treatment, indicating that RAS has the potential to enhance glycolysis, strengthen the energy cycle, and bolster energy provision in the body. Galabiosylceramide, a ceramide dihexoside, is derived from sphingosine and is subject to degradation by α-galactosidase A in lysosomes, resulting in X-linked lysosomal storage disorder (Kamzolova et al., 2003). Notably, sphingosine is involved in cancer cell apoptosis via stimulation of phospholipase C-protein kinase C and activation of phospholipase D, serving as a metabolic marker for metabolic and chronic diseases (Dowdy et al., 2020; Akiyama et al., 2021).

The increase in galabiosylceramide content in BD rats post-treatment suggests that RAS may effectively treat metabolic diseases associated with BD syndrome by regulating sphingolipid metabolism. Arachidonic acid metabolism is a core pathway in inflammatory metabolism, exerting a significant influence on immune function (Wang et al., 2020). Under the influence of lipoxygenase, this pathway leads to the production of immunomodulatory factors such as prostaglandin E2 (PGE2) and leukotriene B4 (LTB4). LTB4 finally forms the metabolite LTE4 via leukotriene C4 synthase and protease processing (Li et al., 2014). Prostaglandin E1 (PGE1), in particular, increases the content of cAMP in vascular smooth muscle cells, leading to vasodilation (Löfgren et al., 2020); enhances the deformability of RBC; activates immunoreactive cells (Amberger et al., 2019); and inhibits platelet aggregation activity, thereby avoiding the formation of AS plaques, reducing the incidence of AS, and providing protection from ischemic myocardial cell injury (Gu et al., 1985). The enhancement of arachidonic acid metabolism in BD rats, as indicated by the increase in endoperoxide G2 and PA (LTE4/i-15:0), gives rise to inflammatory reactions. RAS, however, can regulate arachidonic acid metabolism, inhibiting the release of inflammatory factors, regulating the production of immune regulatory factors, and reducing the body’s inflammatory response. The elevation of PA (i-19:0/PGE1) suggests an increase in production of PGE1, which effectively inhibits platelet aggregation, enhances erythrocyte deformation ability, and holds promise for the effective treatment of BD syndrome. Furthermore, some researchers have identified a connection between RAS and the regulation of ketone synthesis, degradation, and pyruvate metabolism, findings that align with the results of this study corroborating the blood-enriching mechanisms of RAS.

The downstream metabolites of PUFA in the body include AA, prostaglandins (PG), thromboxane, interleukin leukotrienes (LTs), and various other inflammatory factors (Chemello et al., 2022). Linolenic acid, an essential fatty acid in the body, is a precursor for prostaglandin synthesis (Liu et al., 2023). Under the action of desaturase and elongation enzymes, α-linolenic acid is gradually converted to linolenic acid. Eicosapentaenoic acid (EPA) and docosahexaenoic acid (DHA) can produce a series of specific anti-inflammatory mediators through the catalysis of cyclooxygenases, lipoxygenases, and cytochrome P450 oxidases, which have significant physiological effects on the body (Li et al., 2006). α-linolenic acid exhibits an inhibitory effect on lipoprotein synthesis in the liver and enhances metabolic rates, contributing to improved resistance to thrombosis. The increase in α-linolenic acid content after treatment indicates that RAS effectively exerts anti-inflammatory effects by influencing linolenic acid metabolism. Moreover, EPA, catalyzed by cyclooxygenases (COX), produces thromboxane A3, which inhibits platelet aggregation and prostacyclin I3, promoting vasodilation, PGE3, and leukotriene LT5, with anti-inflammatory properties (Anand and Kaithwas, 2014). Linoleic acid protects cardiovascular and cerebrovascular processes, cell membrane synthesis, cholesterol synthesis, and metabolism. In its absence, the body experiences cholesterol metabolism disorders, leading to gradual deposition of cholesterol in the vascular walls, ultimately resulting in atherosclerosis (AS). Accumulated PUFA, on the other hand, primarily serves to prevent AS. RAS effectively reduces the accumulation of PUFA and thus prevents AS. By regulating energy metabolism, linoleic acid inhibits fat deposition in mice (Cao et al., 2018). The increase in linoleic acid content post-treatment signifies that RAS can impact energy metabolism by regulating linoleic acid content, addressing BD syndrome.

**FIGURE 12:**
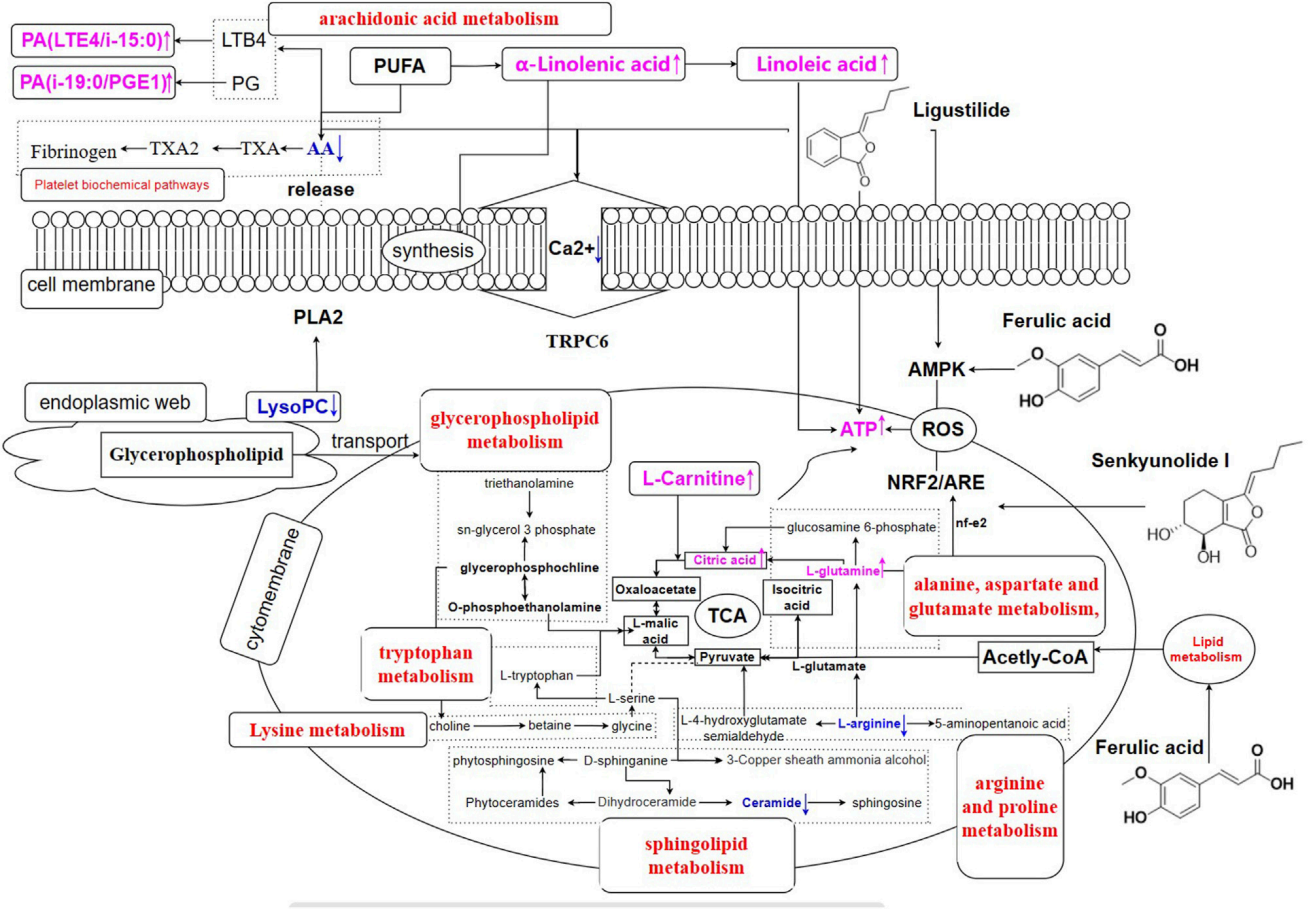
Regulation on metabolic network of RAS in BD rats (control vs. model group, the purple towards up-regulating; the blue towards down-regulating).

The spleen is a primary immune organ. RAS can regulate the levels of glycerophospholipids in the spleen, enhance the body’s immune system, and effectively fight inflammation. Cyclophosphamide induces immune inhibition through lipid metabolism disorder (Simpson et al., 2021). Lipid metabolism disorders are one of the factors that induce AS. RAS can regulate the increase in TG content that occurs in the spleen of BD rats and regulate lipid metabolism, which returns to normal, indicating that RAS can effectively prevent AS, thus reducing the probability of diseases such as myocardial infarction and stroke. In contrast, when the body is in an ischemic state, the spleen can release a large number of monocytes into the plasma, activate the angiotensin II signaling pathway (Li et al., 2015), induce cell migration to the heart, kidney, and other organs, and maintain the everyday activities of the body (Leuschner et al., 2010). Conversely, several marrow progenitor cells migrate out of the bone marrow and accumulate in the spleen, resulting in extramedullary hematopoietic function. Simultaneously, several red blood cells accumulate in the spleen, aggravating anemia and resulting in a significant increase in the spleen index and splenomegaly (Shen, 2016). As an important source of inflammatory cells, the spleen restores lipid metabolism and enhances immunity. RAS can treat bone marrow perivascular mesenchymal progenitor cell damage and enhance the effects on the blood (Zhang et al., 2019b), restoring splenomegaly. The potential mechanism of action of RAS in the treatment of blood-deficient rats is shown in Figure 12.

## 5 Conclusion

In this study, we have extensively explored the attributive classification of various grades of RAS through a combination of *in vivo* and *in vitro* assessments. We have also investigated the therapeutic mechanisms underpinning the effectiveness of RAS in replenishing and invigorating blood circulation. To the best of our knowledge, this research marks the first attempt to elucidate the bioactive features associated with different grades of RAS. Our key findings reveal that the third grade of RAS exhibits a significantly superior capacity to replenish and invigorate blood circulation. Furthermore, we have identified ferulic acid, ligustilide, senkyunolide I, uridine, and guanine as valuable Q-markers for assessment and and enhancement of the blood circulation effects of RAS. The metabolic pathways investigated in our study, including glycerophospholipid metabolism, sphingolipid metabolism, arachidonic acid metabolism, the TCA cycle, and amino acid metabolism, form the core avenues through which RAS addresses BD syndrome. This intricate understanding of metabolic interactions and the impact of RAS within these pathways has provided valuable insights into the relevant therapeutic mechanisms. Moreover, our findings suggest that RAS may be crucial in restoring lipid metabolism. It achieves this by effectively regulating glycerophospholipid and sphingolipid metabolic processes, optimizing the TCA cycle and amino acid metabolism, and ultimately enhancing energy metabolism. The regulation of arachidonic acid metabolism further promotes blood circulation, addressing the core issues associated with BD. In summary, this study has substantially contributed to the scientific classification of commercial grades of RAS, which holds significant promise in TCM. As the research field evolves, our findings offer a reasonable methodological reference for the establishment of standardized grading in TCM.

## Data Availability

The original contributions presented in the study are included in the article/Supplementary Material, further inquiries can be directed to the corresponding author.
