# Supplementary material for: Anti-platelet aggregation activities of different grades of Angelica sinensis and their therapeutic mechanisms in rats with blood deficiency: insights from metabolomics and lipidomics analyses
Source: Front Pharmacol. 2024 Jan 3;14:1230861. doi: 10.3389/fphar.2023.1230861 (PMC10791921; doi:10.3389/fphar.2023.1230861)
Supplement: Supplementary file 1 [file DataSheet1.docx]

Supplementary Material

Anti-platelet aggregation activities of different grades of *Angelica sinensis* and their therapeutic mechanisms in rats with blood deficiency: Insights from metabolomics and lipidomics analyses

**Xue Shen^1^, Yangyang Wu^1^, Ping Chen^1^, Yuwei Bai^1^, Yanan Liu^1^, Yihan Jiang^1^,** **Yawen Zhang^1,2^, Zhigang Yang^1,2,3*^**

^1^School of Pharmacy, Lanzhou University, Lanzhou 730000, China

^2^Collaborative Innovation Center for Northwestern Chinese Medicine, Lanzhou University, Lanzhou 730000, China

^3^State Key Laboratory of Applied Organic Chemistry, Lanzhou University, Lanzhou 730000, China

*** Correspondence:** Zhigang Yang, yangzg@lzu.edu.cn

#
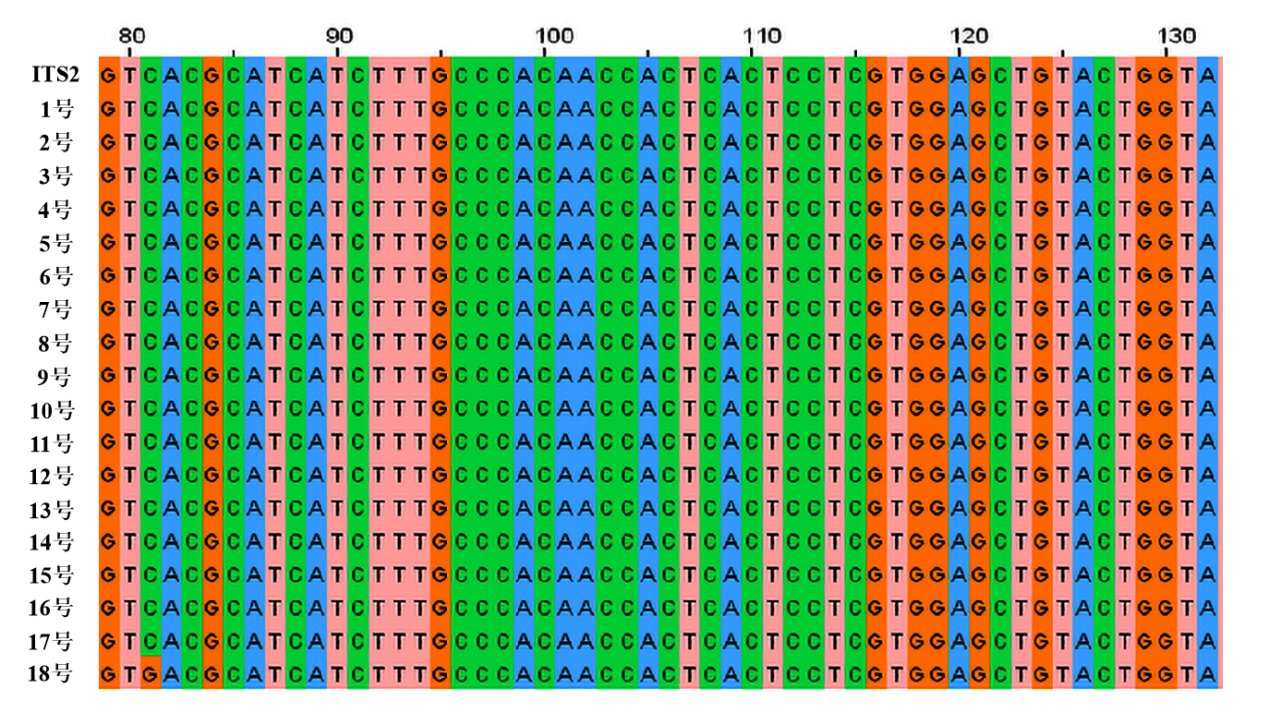
Supplementary Figures

**Supplementary Figure 1.** The result of PCR tests to identify RAS.

**
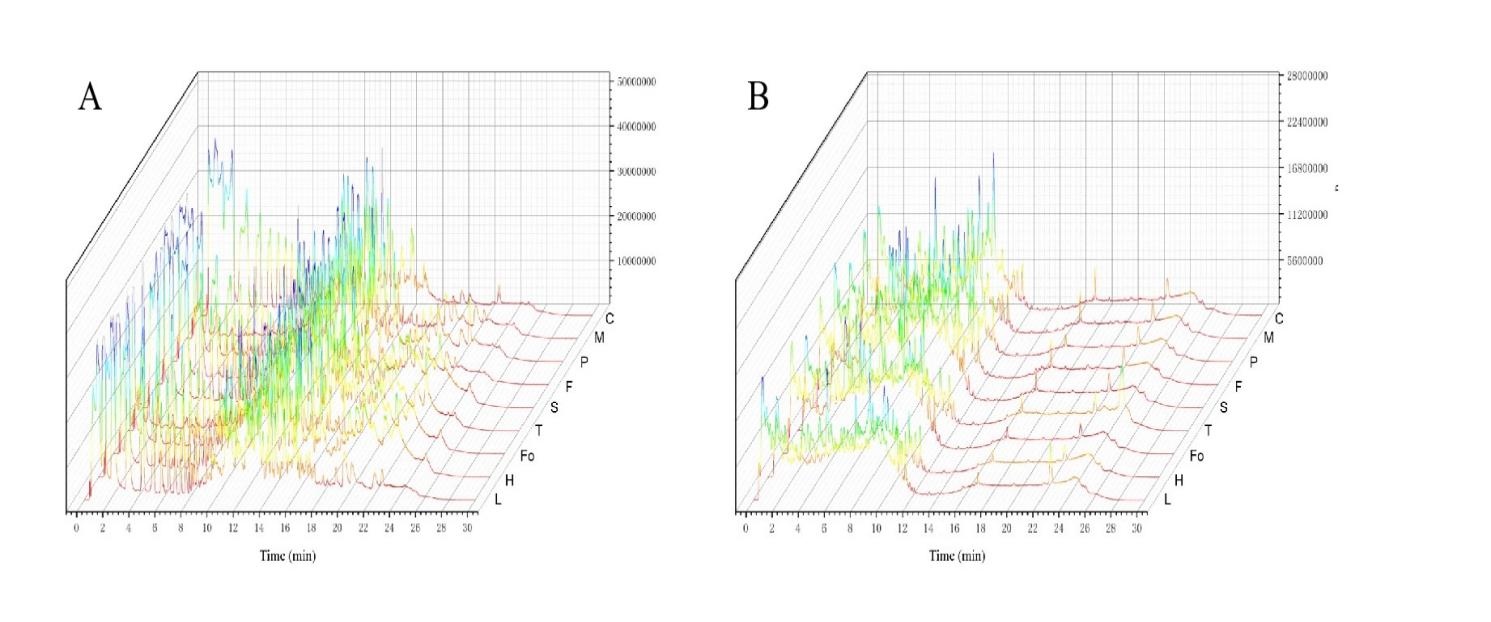
**

**Supplementary Figure 2.** The TIC of plasma (A) and urine (B) of different groups by UPLC-QTOF-MS in positive mode.


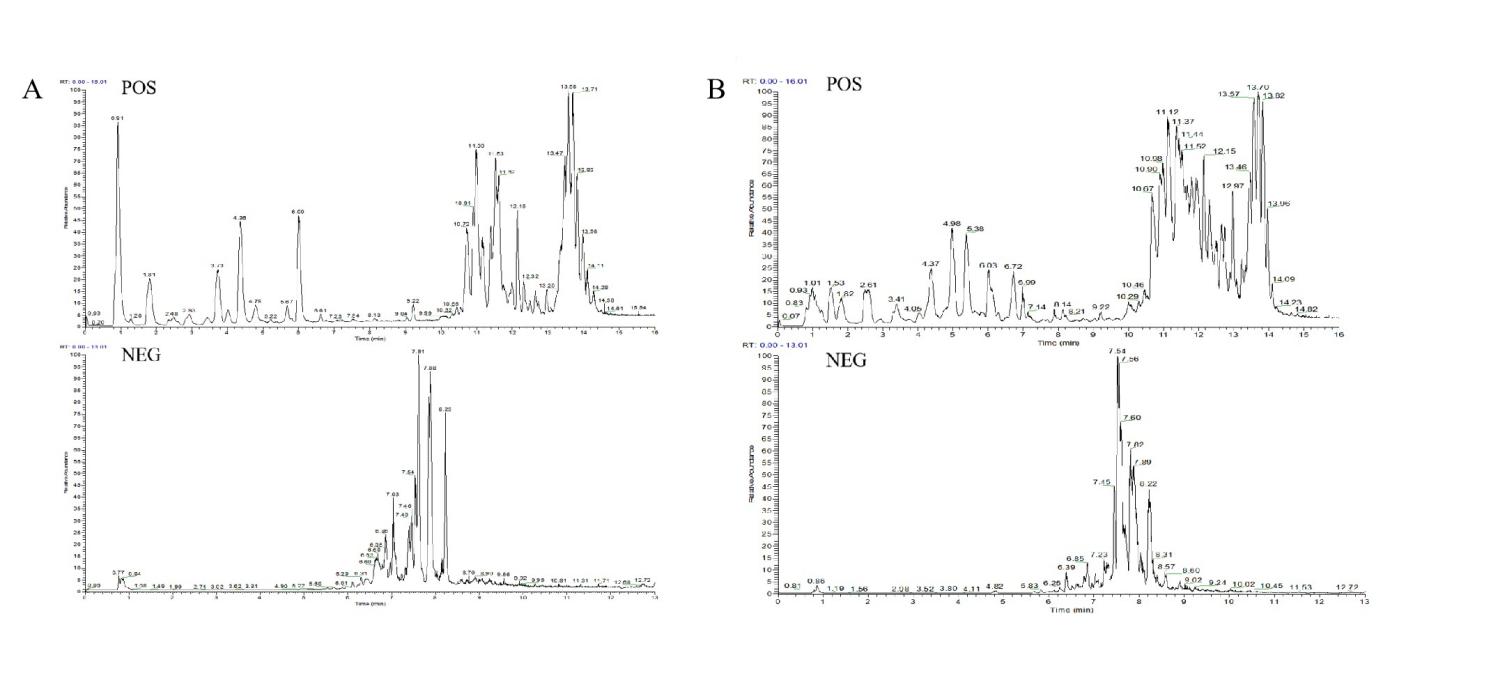


**Supplementary Figure 3.** The TIC of plasma (A) and spleen (B) of different groups by UPLC-Q Exactive MS.

# Supplementary Tables

**Supplementary Table 1.** The result of methodological investigation of HPLC.

|  | relative retention time | the average peak area |
| --- | --- | --- |
| precision | 0.03%~0.52% | 0.73%~3.42% |
| Repeatability | 0.04%~0.77% | 0.21%~3.57% |
| Sample stability | 0~0.69% | 0~5.14% |
| 24h stabilization | 0~1.49% | 0~1.44% |

**Supplementary Table 2.** Summary of various Chemometric models used in the analysis of UPLC-QTOF-MS spectra of plasma and urine samples.

| Parameter of study | Chemometric model | Number of components | R^2^X (cum) | R^2^Y (cum) | Q^2^ (cum) |
| --- | --- | --- | --- | --- | --- |
| Plasma of model | OPLS-DA | 3 | - | 0.668 | 0.713 |
| Plasma of grade | OPLS-DA | 3 | - | 0.853 | 0.507 |
| Urine of model | OPLS-DA | 3 | - | 0.806 | 0.742 |
| Urine of grade | OPLS-DA | 3 | - | 0.853 | 0.560 |

**Supplementary Table 3.** Summary of various Chemometric models used in the analysis of UPLC-Q Exactive MS spectra of plasma and spleen samples.

| Parameter of study | Chemometric model | Number of components | R^2^X (cum) | R^2^Y (cum) | Q^2^ (cum) |
| --- | --- | --- | --- | --- | --- |
| Plasma of POS | PCA | 3 | 0.766 | - | 0.428 |
| Plasma of NEG | PCA | 4 | 0.734 | - | 0.377 |
| Spleen of POS | PCA | 6 | 0.638 | - | 0.448 |
| Spleen of NEG | PCA | 6 | 0.638 | - | 0.471 |
| Plasma of POS | OPLS-DA | 2 | - | 0.976 | 0.845 |
| Plasma of NEG | OPLS-DA | 2 | - | 0.992 | 0.923 |
| Spleen of POS | OPLS-DA | 2 | - | 0.992 | 0.934 |
| Spleen of NEG | OPLS-DA | 2 | - | 0.997 | 0.966 |

**Supplementary Table 4.** The identification information of differential endogenous lipids in plasma of BD rats

| No. | TR (time) | Observed m/z | Adduct | Mode | VIP | Molecular Formula | Metabolites |
| --- | --- | --- | --- | --- | --- | --- | --- |
| 1 | 11.15 | 860.6136 | M+H | POS | 2.34 | C_50_H_86_NO_8_P | PC(22:2/20:5) |
| 2 | 7.18 | 275.2013 | M-H | NEG | 2.16 | C_18_H_28_O_2_ | Stearidonic acid |
| 3 | 4.36 | 546.3545 | M+H | POS | 2.15 | C_28_H_52_NO_7_P | LysoPC(20:3/0:0) |
| 4 | 5.21 | 548.3708 | M+H | POS | 2.06 | C_28_H_54_NO_7_P | LysoPC(20:2/0:0) |
| 5 | 2.88 | 542.3237 | M+H | POS | 1.94 | C_28_H_48_NO_7_P | LysoPC(20:5/0:0) |
| 6 | 4.10 | 570.3550 | M+H | POS | 1.92 | C_30_H_52_NO_7_P | LysoPC(22:5/0:0) |
| 7 | 10.91 | 764.5217 | M+H | POS | 1.84 | C_43_H_74_NO_8_P | PE(22:6/16:0) |
| 8 | 7.58 | 329.2481 | M-H | NEG | 1.81 | C_22_H_34_O_2_ | 4,7,10,13,16-docosapentaenoic acid |
| 9 | 7.32 | 301.2168 | M-H | NEG | 1.80 | C_20_H_30_O_2_ | 2*E*,5*Z*,8*Z*,11*Z*,14*Z*-eicosapentaenoic acid |
| 10 | 7.37 | 277.2167 | M-H | NEG | 1.74 | C_18_H_30_O_2_ | alpha-Linolenic acid |
| 11 | 7.71 | 305.2482 | M-H | NEG | 1.73 | C_20_H_34_O_2_ | 5,8,11-eicosatrienoic acid |
| 12 | 6.26 | 550.3862 | M+H | POS | 1.71 | C_28_H_56_NO_7_P | LysoPC(20:1/0:0) |
| 13 | 8.07 | 333.2797 | M-H | NEG | 1.67 | C_22_H_38_O_2_ | 3*E*,13*Z*,16*Z*-Docosatrienoic acid |
| 14 | 11.69 | 745.6218 | M+H | POS | 1.62 | C_42_H_85_N_2_O_6_P | SM(d18:1/19:0) |
| 15 | 7.96 | 307.2639 | M-H | NEG | 1.61 | C_20_H_36_O_2_ | 8,11-Eicosadienoic acid |
| 16 | 11.05 | 790.5724 | M+H | POS | 1.57 | C_46_H_80_NO_7_P | PC(P-18:1/20:5) |
| 17 | 3.15 | 494.3239 | M+H | POS | 1.55 | C_24_H_48_NO_7_P | LysoPC(16:1/0:0) |
| 18 | 11.51 | 783.6373 | M+H | POS | 1.53 | C_45_H_87_N_2_O_6_P | SM(d18:2/22:1) |
| 19 | 7.52 | 253.2167 | M-H | NEG | 1.51 | C_16_H_30_O_2_ | 7*Z*-palmitoleic acid |
| 20 | 12.54 | 798.6814 | M+H | POS | 1.51 | C_47_H_91_NO_8_ | GalCer(d18:1/23:0) |
| 21 | 11.17 | 716.5218 | M+H | POS | 1.49 | C_39_H_74_NO_8_P | PE(18:2/16:0) |
| 22 | 7.81 | 331.2639 | M-H | NEG | 1.48 | C_22_H_36_O_2_ | Adrenic acid |
| 23 | 11.54 | 792.5547 | M+H | POS | 1.47 | C_45_H_78_NO_8_P | PE(18:0/22:6) |
| 24 | 10.63 | 740.5572 | M+H | POS | 1.44 | C_42_H_78_NO_7_P | PC(P-16:0/18:3) |
| 25 | 12.40 | 784.6658 | M+H | POS | 1.43 | C_46_H_89_NO_8_ | GalCer(d18:1/22:0) |

| No. | TR (time) | Observed m/z | Adduct | Mode | VIP | Molecular Formula | Metabolites |
| --- | --- | --- | --- | --- | --- | --- | --- |
| 26 | 11.16 | 792.5880 | M+H | POS | 1.41 | C_46_H_82_NO_7_P | PC(P-18:0/20:5) |
| 27 | 11.76 | 812.6141 | M+H | POS | 1.40 | C_46_H_86_NO_8_P | PC(18:0/20:3) |
| 28 | 8.23 | 911.5651 | M-H | NEG | 1.39 | C_49_H_85_O_13_P | PI(18:1/22:4) |
| 29 | 8.24 | 309.2795 | M-H | NEG | 1.39 | C_20_H_38_O_2_ | 8*Z*-eicosenoic acid |
| 30 | 2.67 | 468.3083 | M+H | POS | 1.37 | C_22_H_46_NO_7_P | LysoPC(14:0/0:0) |
| 31 | 11.22 | 766.5731 | M+H | POS | 1.36 | C_44_H_80_NO_7_P | PC(P-16:0/20:4) |
| 32 | 4.79 | 522.3548 | M+H | POS | 1.36 | C_26_H_52_NO_7_P | LysoPC(18:1/0:0) |
| 33 | 8.90 | 365.3419 | M-H | NEG | 1.35 | C_24_H_46_O_2_ | 19*Z*-Tetracosenoic acid |
| 34 | 11.75 | 820.6195 | M+H | POS | 1.34 | C_48_H_86_NO_7_P | PC(P-18:0/22:5) |
| 35 | 3.75 | 544.3385 | M+H | POS | 1.33 | C_28_H_50_NO_7_P | LysoPC(20:4/0:0) |
| 36 | 7.71 | 267.2326 | M-H | NEG | 1.32 | C_17_H_32_O_2_ | 9*E*-heptadecenoic acid |
| 37 | 12.39 | 810.6799 | M+H | POS | 1.32 | C_48_H_91_NO_8_ | GalCer(d18:1/24:1) |
| 38 | 8.82 | 606.4490 | M+H | POS | 1.32 | C_32_H_64_NO_7_P | LysoPC(24:1/0:0) |
| 39 | 12.66 | 812.6971 | M+H | POS | 1.31 | C_48_H_93_NO_8_ | GalCer(d18:1/24:0) |
| 40 | 13.56 | 846.7529 | M+NH_4_ | POS | 1.30 | C_53_H_96_O_6_ | TG(14:0/16:0/20:3) |
| 41 | 8.30 | 913.5809 | M-H | NEG | 1.29 | C_49_H_87_O_13_P | PI(18:0/22:4) |
| 42 | 4.95 | 572.3708 | M+H | POS | 1.27 | C_30_H_54_NO_7_P | LysoPC(22:4/0:0) |
| 43 | 7.90 | 281.2481 | M-H | NEG | 1.27 | C_18_H_34_O_2_ | Oleic acid |
| 44 | 13.88 | 838.7848 | M+NH_4_ | POS | 1.24 | C_52_H_100_O_6_ | TG(15:0/16:0/18:0) |
| 45 | 3.47 | 482.3237 | M+H | POS | 1.24 | C_23_H_48_NO_7_P | LysoPC(15:0/0:0) |
| 46 | 12.21 | 866.6624 | M+H | POS | 1.23 | C_50_H_92_NO_8_P | PC(22:1/20:3) |
| 47 | 12.63 | 841.7146 | M+H | POS | 1.23 | C_49_H_97_N_2_O_6_P | SM(d18:1/26:1) |
| 48 | 14.26 | 694.6490 | M+NH_4_ | POS | 1.23 | C_47_H_80_O_2_ | CE(20:2) |
| 49 | 12.31 | 824.6500 | M+H | POS | 1.21 | C_48_H_90_NO_7_P | PC(P-18:1/22:2) |
| 50 | 13.75 | 810.7528 | M+NH_4_ | POS | 1.18 | C_50_H_96_O_6_ | TG(15:0/16:0/16:0) |
| 51 | 11.09 | 740.5219 | M+H | POS | 1.17 | C_41_H_74_NO_8_P | PE(20:4/16:0) |
| No. | TR (time) | Observed m/z | Adduct | Mode | VIP | Molecular Formula | Metabolites |
| 52 | 3.74 | 520.3389 | M+H | POS | 1.17 | C_26_H_50_NO_7_P | LysoPC(18:2/0:0) |
| 53 | 8.08 | 871.5340 | M-H | NEG | 1.16 | C_46_H_81_O_13_P | PI(15:0/22:4) |
| 54 | 3.67 | 568.3387 | M+H | POS | 1.16 | C_30_H_50_NO_7_P | LysoPC(22:6/0:0) |
| 55 | 11.72 | 718.5737 | M+H | POS | 1.15 | C_40_H_80_NO_7_P | PC(O-16:0/16:1) |
| 56 | 12.46 | 868.6782 | M+H | POS | 1.15 | C_50_H_94_NO_8_P | PC(20:1/22:2) |
| 57 | 11.78 | 822.6341 | M+H | POS | 1.15 | C_48_H_88_NO_7_P | PC(P-18:0/22:4) |
| 58 | 7.61 | 279.2324 | M-H | NEG | 1.14 | C_18_H_32_O_2_ | Linoleic acid |
| 59 | 12.04 | 814.6306 | M+H | POS | 1.11 | C_46_H_88_NO_8_P | PC(18:0/20:2) |
| 60 | 10.80 | 832.5817 | M+H | POS | 1.11 | C_48_H_82_NO_8_P | PC(20:4/20:3) |
| 61 | 11.83 | 746.6049 | M+H | POS | 1.10 | C_42_H_84_NO_7_P | PC(O-16:0/18:1) |
| 62 | 11.68 | 768.5534 | M+H | POS | 1.10 | C_43_H_78_NO_8_P | PE(20:4/18:0) |
| 63 | 10.96 | 808.5819 | M+H | POS | 1.09 | C_46_H_82_NO_8_P | PC(20:4/18:1) |
| 64 | 11.67 | 848.6139 | M+Na | POS | 1.07 | C_47_H_88_NO_8_P | PE(20:3/22:0) |
| 65 | 11.21 | 818.6026 | M+H | POS | 1.06 | C_48_H_84_NO_7_P | PC(P-18:0/22:6) |
| 66 | 12.38 | 800.6507 | M+H | POS | 1.05 | C_46_H_90_NO_7_P | PC(P-18:0/20:1) |
| 67 | 11.39 | 794.6040 | M+H | POS | 1.04 | C_46_H_84_NO_7_P | PC(P-18:0/20:4) |
| 68 | 11.17 | 766.5367 | M+H | POS | 1.03 | C_43_H_76_NO_8_P | PE(20:5/18:0) |
| 69 | 5.21 | 510.3553 | M+H | POS | 1.03 | C_25_H_52_NO_7_P | LysoPE(20:0/0:0) |
| 70 | 11.46 | 656.5236 | M+NH_4_ | POS | 1.02 | C_41_H_66_O_5_ | DG(18:2/20:5/0:0) |
| 71 | 11.57 | 836.6132 | M+H | POS | 1.02 | C_48_H_86_NO_8_P | PC(18:0/22:5) |
| 72 | 11.35 | 768.5881 | M+H | POS | 1.02 | C_44_H_82_NO_7_P | PC(P-18:0/18:3) |
| 73 | 13.81 | 824.7689 | M+NH_4_ | POS | 1.00 | C_51_H_98_O_6_ | TG(16:0/16:0/16:0) |

**Supplementary Table 5.** The identification information of differential endogenous lipids in spleen of BD rats.

| No. | TR (time) | Observed m/z | Adduct | Mode | VIP | Molecular Formula | Metabolites |
| --- | --- | --- | --- | --- | --- | --- | --- |
| 1 | 5.24 | 548.3716 | M+H | POS | 2.08 | C_28_H_54_NO_7_P | LysoPC(20:2/0:0) |
| 2 | 6.28 | 550.3867 | M+H | POS | 2.09 | C_28_H_56_NO_7_P | LysoPC(20:1/0:0) |
| 3 | 6.40 | 508.3408 | M+H | POS | 2.07 | C_25_H_50_NO_7_P | PE(20:1/0:0) |
| 4 | 13.43 | 844.7377 | M+NH_4_ | POS | 2.06 | C_53_H_94_O_6_ | TG(14:0/16:0/20:4) |
| 5 | 4.97 | 572.3705 | M+H | POS | 2.02 | C_30_H_54_NO_7_P | LysoPC(22:4/0:0) |
| 6 | 13.46 | 870.7533 | M+NH_4_ | POS | 2.01 | C_55_H_96_O_6_ | TG(16:0/16:1/20:4) |
| 7 | 11.15 | 860.6141 | M+H | POS | 1.97 | C_50_H_86_NO_8_P | PC(22:2/20:5) |
| 8 | 4.36 | 546.3548 | M+H | POS | 1.97 | C_28_H_52_NO_7_P | LysoPC(20:3/0:0) |
| 9 | 7.63 | 578.4181 | M+H | POS | 1.97 | C_30_H_60_NO_7_P | LysoPC(22:1/0:0) |
| 10 | 13.51 | 858.7543 | M+NH_4_ | POS | 1.94 | C_54_H_96_O_6_ | TG(15:0/16:0/20:4) |
| 11 | 3.76 | 520.3399 | M+H | POS | 1.92 | C_26_H_50_NO_7_P | LysoPC(18:2/0:0) |
| 12 | 12.10 | 840.6447 | M+H | POS | 1.86 | C_48_H_90_NO_8_P | PC(20:0/20:3) |
| 13 | 14.03 | 690.6180 | M+NH_4_ | POS | 1.86 | C_47_H_76_O_2_ | CE(20:4) |
| 14 | 11.72 | 682.5398 | M+H | POS | 1.85 | C_43_H_68_O_5_ | DG(18:2/22:6/0:0) |
| 15 | 4.85 | 480.3098 | M+H | POS | 1.83 | C_23_H_46_NO_7_P | PE(18:1/0:0) |
| 16 | 13.56 | 846.7533 | M+NH_4_ | POS | 1.83 | C_53_H_96_O_6_ | TG(14:0/16:0/20:3) |
| 17 | 10.79 | 770.5671 | M+H | POS | 1.80 | C_43_H_80_NO_8_P | PC(15:0/20:3) |
| 18 | 12.21 | 866.6619 | M+H | POS | 1.76 | C_50_H_92_NO_8_P | PC(22:1/20:3) |
| 19 | 4.81 | 522.3551 | M+H | POS | 1.76 | C_26_H_52_NO_7_P | LysoPC(18:1/0:0) |
| 20 | 12.44 | 868.6768 | M+H | POS | 1.76 | C_50_H_94_NO_8_P | PC(20:1/22:2) |
| 21 | 10.50 | 756.5525 | M+H | POS | 1.75 | C_42_H_78_NO_8_P | PC(18:3/16:0) |
| 22 | 3.71 | 478.2930 | M+H | POS | 1.75 | C_23_H_44_NO_7_P | PE(18:2/0:0) |
| 23 | 12.04 | 864.6461 | M+H | POS | 1.74 | C_50_H_90_NO_8_P | PC(22:1/20:4) |

| No. | TR (time) | Observed m/z | Adduct | Mode | VIP | Molecular Formula | Metabolites |
| --- | --- | --- | --- | --- | --- | --- | --- |
| 24 | 13.65 | 860.7695 | M+NH_4_ | POS | 1.71 | C_54_H_98_O_6_ | TG(15:0/16:0/20:3) |
| 25 | 13.51 | 832.7388 | M+NH_4_ | POS | 1.71 | C_52_H_94_O_6_ | TG(15:0/16:0/18:3) |
| 26 | 13.54 | 820.7379 | M+NH_4_ | POS | 1.70 | C_51_H_94_O_6_ | TG(16:0/16:1/16:1) |
| 27 | 13.58 | 872.7682 | M+NH_4_ | POS | 1.68 | C_55_H_98_O_6_ | TG(16:0/16:0/20:4) |
| 28 | 10.09 | 704.5214 | M+H | POS | 1.67 | C_38_H_74_NO_8_P | PC(16:1/14:0) |
| 29 | 10.27 | 742.5366 | M+H | POS | 1.62 | C_41_H_76_NO_8_P | PC(15:1/18:2) |
| 30 | 13.69 | 848.7691 | M+NH_4_ | POS | 1.61 | C_53_H_98_O_6_ | TG(16:0/16:0/18:2) |
| 31 | 11.11 | 834.5984 | M+H | POS | 1.60 | C_48_H_84_NO_8_P | PC(18:0/22:6) |
| 32 | 13.82 | 850.7848 | M+NH_4_ | POS | 1.59 | C_53_H_100_O_6_ | TG(16:0/16:0/18:1) |
| 33 | 12.35 | 816.6464 | M+H | POS | 1.57 | C_46_H_90_NO_8_P | PC(20:0/18:1) |
| 34 | 8.14 | 538.4229 | M+H | POS | 1.57 | C_28_H_60_NO_6_P | PC(O-20:0/0:0) |
| 35 | 13.67 | 822.7535 | M+NH_4_ | POS | 1.57 | C_51_H_96_O_6_ | TG(16:0/16:0/16:1) |
| 36 | 10.28 | 730.5368 | M+H | POS | 1.56 | C_40_H_76_NO_8_P | PC(14:0/18:2) |
| 37 | 8.84 | 606.4491 | M+H | POS | 1.56 | C_32_H_64_NO_7_P | LysoPC(24:1/0:0) |
| 38 | 10.32 | 830.5677 | M+H | POS | 1.55 | C_48_H_80_NO_8_P | PC(20:4/20:4) |
| 39 | 10.18 | 754.5369 | M+H | POS | 1.55 | C_42_H_76_NO_8_P | PC(14:0/20:4) |
| 40 | 3.19 | 494.3242 | M+H | POS | 1.55 | C_24_H_48_NO_7_P | LysoPC(16:1/0:0) |
| 41 | 13.77 | 862.7850 | M+NH_4_ | POS | 1.54 | C_54_H_100_O_6_ | TG(15:0/16:0/20:2) |
| 42 | 3.48 | 544.3394 | M+H | POS | 1.53 | C_28_H_50_NO_7_P | LysoPC(20:4/0:0) |
| 43 | 10.64 | 744.5517 | M+H | POS | 1.53 | C_41_H_78_NO_8_P | PC(15:0/18:2) |
| 44 | 10.63 | 740.5568 | M+H | POS | 1.53 | C_42_H_78_NO_7_P | PC(P-16:0/18:3) |
| 45 | 3.48 | 482.3240 | M+H | POS | 1.53 | C_23_H_48_NO_7_P | LysoPC(15:0/0:0) |
| 46 | 10.95 | 705.5892 | M+H | POS | 1.53 | C_39_H_81_N_2_O_6_P | SM(d18:0/16:0) |
| 47 | 12.05 | 814.6306 | M+H | POS | 1.52 | C_46_H_88_NO_8_P | PC(18:0/20:2) |
| 48 | 13.71 | 874.7843 | M+NH_4_ | POS | 1.50 | C_55_H_100_O_6_ | TG(16:0/16:0/20:3) |
| 49 | 12.48 | 594.5817 | M+H | POS | 1.49 | C_38_H_75_NO_3_ | Cer(d18:1/20:0) |
| No. | TR (time) | Observed m/z | Adduct | Mode | VIP | Molecular Formula | Metabolites |
| 50 | 12.39 | 842.6615 | M+H | POS | 1.48 | C_48_H_92_NO_8_P | PC(20:0/20:2) |
| 51 | 13.83 | 876.8000 | M+NH_4_ | POS | 1.46 | C_55_H_102_O_6_ | TG(16:0/18:0/18:2) |
| 52 | 2.69 | 468.3077 | M+H | POS | 1.45 | C_22_H_46_NO_7_P | LysoPC(14:0/0:0) |
| 53 | 13.63 | 834.7536 | M+NH_4_ | POS | 1.43 | C_52_H_96_O_6_ | TG(15:0/16:0/18:2) |
| 54 | 13.81 | 824.7692 | M+NH_4_ | POS | 1.42 | C_51_H_98_O_6_ | TG(16:0/16:0/16:0) |
| 55 | 12.78 | 870.6936 | M+H | POS | 1.40 | C_50_H_96_NO_8_P | PC(20:0/22:2) |
| 56 | 9.99 | 778.5364 | M+H | POS | 1.38 | C_44_H_76_NO_8_P | PC(22:6/14:0) |
| 57 | 4.28 | 506.3597 | M+H | POS | 1.37 | C_26_H_52_NO_6_P | LysoPC(P-18:1/0:0) |
| 58 | 5.22 | 510.3553 | M+H | POS | 1.35 | C_25_H_52_NO_7_P | LysoPE(20:0/0:0) |
| 59 | 12.22 | 636.5557 | M+NH_4_ | POS | 1.32 | C_39_H_70_O_5_ | DG(16:0/20:3/0:0) |
| 60 | 4.38 | 454.2923 | M+H | POS | 1.31 | C_21_H_44_NO_7_P | PE(16:0/0:0) |
| 61 | 11.75 | 812.6144 | M+H | POS | 1.31 | C_46_H_86_NO_8_P | PC(18:0/20:3) |
| 62 | 11.11 | 784.5831 | M+H | POS | 1.31 | C_44_H_82_NO_8_P | PC(20:3/16:0) |
| 63 | 12.04 | 684.5555 | M+NH_4_ | POS | 1.30 | C_43_H_70_O_5_ | DG(18:2/22:5/0:0) |
| 64 | 7.01 | 599.3199 | M-H | NEG | 1.30 | C_27_H_53_O_12_P | PI(18:0/0:0) |
| 65 | 9.25 | 367.3575 | M-H | NEG | 1.30 | C_24_H_48_O_2_ | Lignoceric acid |
| 66 | 12.07 | 746.5696 | M+H | POS | 1.30 | C_41_H_80_NO_8_P | PE(18:0/18:1) |
| 67 | 11.58 | 836.6134 | M+H | POS | 1.29 | C_48_H_86_NO_8_P | PC(18:0/22:5) |
| 68 | 9.86 | 675.5429 | M+H | POS | 1.29 | C_37_H_75_N_2_O_6_P | SM(d18:1/14:0) |
| 69 | 12.64 | 844.6773 | M+H | POS | 1.28 | C_48_H_94_NO_8_P | PC(20:0/20:1) |
| 70 | 13.76 | 836.7694 | M+NH_4_ | POS | 1.28 | C_52_H_98_O_6_ | TG(15:0/16:0/18:1) |
| 71 | 10.55 | 768.5526 | M+H | POS | 1.26 | C_43_H_78_NO_8_P | PC(20:4/15:0) |
| 72 | 6.71 | 510.3914 | M+H | POS | 1.25 | C_26_H_56_NO_6_P | PC(O-18:0/0:0) |
| 73 | 3.39 | 526.2926 | M+H | POS | 1.25 | C_27_H_44_NO_7_P | LysoPE(22:6/0:0) |
| 74 | 11.49 | 770.6036 | M+H | POS | 1.25 | C_44_H_84_NO_7_P | PC(P-18:0/18:2) |
| 75 | 4.39 | 496.3391 | M+H | POS | 1.25 | C_24_H_50_NO_7_P | LysoPC(16:0/0:0) |
| 76 | 8.92 | 339.3263 | M-H | NEG | 1.24 | C_22_H_44_O_2_ | Behenic acid |
| No. | TR (time) | Observed m/z | Adduct | Mode | VIP | Molecular Formula | Metabolites |
| 77 | 11.27 | 742.5378 | M+H | POS | 1.24 | C_41_H_76_NO_8_P | PE(18:2/18:1) |
| 78 | 10.29 | 802.5347 | M+H | POS | 1.22 | C_46_H_76_NO_8_P | PC(18:3/20:5) |
| 79 | 11.87 | 800.6144 | M+H | POS | 1.22 | C_45_H_86_NO_8_P | PE(18:2/22:0) |
| 80 | 7.95 | 857.5184 | M-H | POS | 1.21 | C_45_H_79_O_13_P | PI(16:0/20:4) |
| 81 | 7.53 | 552.4025 | M+H | POS | 1.21 | C_28_H_58_NO_7_P | LysoPC(20:0/0:0) |
| 82 | 12.13 | 566.5503 | M+H | POS | 1.21 | C_36_H_71_NO_3_ | Cer(d18:1/18:0) |
| 83 | 6.74 | 466.3289 | M+H | POS | 1.21 | C_23_H_48_NO_6_P | PE(P-18:0/0:0) |
| 84 | 6.13 | 482.3239 | M+H | POS | 1.20 | C_23_H_48_NO_7_P | PE(18:0/0:0) |
| 85 | 11.61 | 820.6192 | M+H | POS | 1.20 | C_48_H_86_NO_7_P | PC(P-18:0/22:5) |
| 86 | 13.31 | 752.6761 | M+NH_4_ | POS | 1.19 | C_46_H_86_O_6_ | TG(14:0/14:0/15:1) |
| 87 | 10.81 | 832.5829 | M+H | POS | 1.19 | C_48_H_82_NO_8_P | PC(20:4/20:3) |
| 88 | 5.38 | 508.3756 | M+H | POS | 1.18 | C_26_H_54_NO_6_P | LysoPC(P-18:0/0:0) |
| 89 | 6.02 | 524.3706 | M+H | POS | 1.18 | C_26_H_54_NO_7_P | LysoPC(18:0/0:0) |
| 90 | 11.33 | 772.5830 | M+H | POS | 1.17 | C_43_H_82_NO_8_P | PE(18:0/20:2) |
| 91 | 11.89 | 772.6196 | M+H | POS | 1.16 | C_44_H_86_NO_7_P | PC(P-18:1/18:0) |
| 92 | 6.82 | 468.3440 | M+H | POS | 1.16 | C_23_H_50_NO_6_P | PE(O-18:0/0:0) |
| 93 | 12.95 | 872.7086 | M+H | POS | 1.15 | C_50_H_98_NO_8_P | PC(20:0/22:1) |
| 94 | 12.19 | 610.5402 | M+NH_4_ | POS | 1.15 | C_37_H_68_O_5_ | DG(16:0/18:2/0:0) |
| 95 | 8.11 | 885.5492 | M-H | POS | 1.14 | C_47_H_83_O_13_P | PI(18:0/20:4) |
| 96 | 11.89 | 838.6299 | M+H | POS | 1.14 | C_48_H_88_NO_8_P | PC(18:0/22:4) |
| 97 | 11.86 | 540.5346 | M+H | POS | 1.13 | C_34_H_69_NO_3_ | Cer(d18:0/16:0) |
| 98 | 10.98 | 808.5826 | M+H | POS | 1.11 | C46H_82_NO_8_P | PC(20:4/18:1) |
| 99 | 3.44 | 502.2927 | M+H | POS | 1.10 | C_25_H_44_NO_7_P | PE(20:4/0:0) |
| 100 | 4.30 | 570.3547 | M+H | POS | 1.10 | C_30_H_52_NO_7_P | LysoPC(22:5/0:0) |
| 101 | 13.67 | 796.7380 | M+NH_4_ | POS | 1.06 | C_49_H_94_O_6_ | TG(14:0/16:0/16:0) |
| 102 | 11.36 | 744.5881 | M+H | POS | 1.06 | C_42_H_82_NO_7_P | PC(P-18:0/16:1) |
| 103 | 11.82 | 776.5572 | M+H | POS | 1.05 | C_45_H_78_NO_7_P | PE(P-18:0/22:6) |
| 104 | 11.10 | 820.5820 | M+H | POS | 1.04 | C_47_H_82_NO_8_P | PE(20:4/22:2) |
| No. | TR (time) | Observed m/z | Adduct | Mode | VIP | Molecular Formula | Metabolites |
| 105 | 5.00 | 438.2973 | M+H | POS | 1.04 | C_21_H_44_NO_6_P | LysoPE(P-16:0/0:0) |
| 106 | 8.03 | 871.5343 | M-H | NEG | 1.04 | C_46_H_81_O_13_P | PI(15:0/22:4) |
| 107 | 12.53 | 628.6022 | M+NH_4_ | POS | 1.04 | C_42_H_74_O_2_ | CE(15:0) |
| 108 | 10.84 | 732.5523 | M+H | POS | 1.04 | C_40_H_78_NO_8_P | PC(14:0/18:1) |
| 109 | 13.18 | 678.6754 | M+H | POS | 1.02 | C_44_H_87_NO_3_ | Cer(d18:1/26:0) |
| 110 | 6.78 | 538.3865 | M+H | POS | 1.01 | C_27_H_56_NO_7_P | PE(22:0/0:0) |
| 111 | 11.98 | 788.6150 | M+H | POS | 1.01 | C_44_H_86_NO_8_P | PC(18:0/18:1) |
| 112 | 11.63 | 786.5994 | M+H | POS | 1.00 | C_44_H_84_NO_8_P | PC(18:0/18:2) |
